# How can we change behavior to bolster biosecurity? A scoping review and recommendations

**DOI:** 10.3389/fvets.2026.1851630

**Published:** 2026-07-22

**Authors:** Julia M. Smith, Jake Fountain, Jennifer Manyweathers, Jenny-Ann Toribio, Suman Das Gupta, Victoria J. Brookes, Marta Hernandez-Jover

**Affiliations:** 1 Department of Animal and Veterinary Sciences, University of Vermont, Burlington, VT, United States; 2 Social Ecological Gaming and Simulation Lab, University of Vermont, Burlington, VT, United States; 3 School of Agricultural, Environmental and Veterinary Sciences, Charles Sturt University, Wagga Wagga, NSW, Australia; 4 Gulbali Institute, Charles Sturt University, Wagga Wagga, NSW, Australia; 5 Sydney School of Veterinary Science, The University of Sydney, Camperdown, NSW, Australia

**Keywords:** behavior change, biosecurity, interventions, livestock disease, scoping review

## Abstract

Farmers’ perceptions and the associated facilitators and barriers relevant to performing biosecurity and animal health measures have been well studied. Few studies, however, have evaluated interventions aiming at promoting the adoption of biosecurity measures. This scoping review was conducted to identify and evaluate such studies. Interventions were mapped to potentially useful frameworks, with the aim of informing the implementation of future biosecurity interventions. Following PRISMA-ScR guidelines, structured literature searches supplemented by reference list screening were conducted. Studies were included if their objective was to evaluate interventions aimed at increasing biosecurity at the farm level to protect livestock from infectious disease challenges. Screening, data extraction, mapping, and conflict resolution were conducted by the first author and at least one co-author. Extracted data on intervention design included context, target participants, desired outcomes, program components, evaluation methods, and theoretical frameworks. Interventions were mapped to behavior change wheel categories, including policies, interventions, and sources of behavior according to the COM-B framework. Of 699 records retrieved, 445 were unique. Of these, 263 underwent full-text screening, and 33 met the inclusion criteria and represented 41 distinct studies conducted or published between 2008 and 2024. Eighteen studies were conducted in the United States or the United Kingdom (12 and 6, respectively), and 25 did not reference a specific disease. Major agricultural species (poultry, swine, cattle, and sheep) were represented, with most studies conducted in commercial production systems (*n* = 25) rather than smallholder or backyard settings (*n* = 8). Outcome indicators most commonly measured behavior (*n* = 31), knowledge (*n* = 15), or attitudes (*n* = 6). Key intervention components were trainings (*n* = 12), surveys (*n* = 7), action plans (*n* = 7), and audits (*n* = 7). Only five studies reported controlled study designs, and fewer than half referenced a theoretical framework. Intervention components most frequently aligned with the behavior change wheel intervention function categories of education, persuasion, enablement, and environmental restructuring. A new function of questioning/observing was proposed. As we characterized these studies of interventions to motivate the adoption of biosecurity measures, we recognized the importance of clearly reporting what was done and why. The application of tools such as intervention mapping and the integration of behavior change frameworks or models in biosecurity implementation studies can help strengthen the evidence base for future biosecurity initiatives.

## 1 Introduction

Whether to reduce the need for treatment with antibiotics or to prevent an epidemic of a new, emerging, or exotic disease, people involved in the care and management of agricultural species are encouraged to implement measures that prevent the introduction and spread of infectious diseases (1). These measures, collectively referred to as biosecurity (2), include a range of practices that can be broadly categorized into access control, hygiene and sanitation, enhancement of immunity, and exposure reduction. The importance of specific measures may vary depending on the disease of concern (3, 4) and the goals of the producer (5, 6). As reviewed by others (7–12), previous studies have sought to understand the perceptions of various types of farmers and the facilitators and barriers relevant to biosecurity and animal health measures in general. Given the complex combination of socio-psychological and economic drivers of behavior, designing and delivering effective interventions to motivate the adoption of biosecurity measures remains challenging. Addressing this challenge requires input from practitioners of multiple disciplines including but not limited to animal and veterinary scientists, social scientists, communication specialists, and marketing experts (7, 12).

The field of health behavior has incorporated behavior change theories and implementation approaches for many years (13). There are many theories of behavior change, which may prove daunting when attempting to link an intervention to theory. Different contexts have produced different theories. Some, like the theory of planned behavior (14) and the health belief model (15), are frequently encountered and have been applied in many fields, including animal health (16–18). These theories are useful for describing determinants or antecedents that predict behavior. These predictors, often referred to as constructs, can be assessed with appropriately designed survey questions. Each question or survey item is linked to a construct, and the strength of its association with behavioral intention or behavior itself can be measured. To stimulate behavior change, an intervention should be designed to target an appropriate antecedent or determinant to achieve the desired outcomes (19).

When designing an intervention, antecedents of more than one theory may be targeted. An understanding of the relevant antecedents, particularly facilitators and barriers, is the key for selecting appropriate techniques to include in the intervention (20). In fact, as Kok et al. (21) point out, behavior change interventions and implementation programs are not limited to focusing on individuals. The context of the desired behavior often determines the level at which the intervention should be targeted. The target could be a group or a community, or more than one level. According to the COM-B framework (19), behavior (B) is influenced by capability (C), opportunity (O), and motivation (M). Each of these sources or antecedents of behavior are further subdivided: capability can be either psychological (i.e., knowledge-based) or physical (i.e., physical skills), opportunity can be either social (e.g., cues or prompts from others) or environmental (e.g., time), and motivation can be reflective (i.e., through cognitive processes or attitudes) or automatic (i.e., through sub-conscious attitudes or habit formation). Dogusan et al. (9) recently reviewed biosecurity intervention literature through the lens of the theoretical domains framework (22, 23), which is related to COM-B. A simpler approach may be to apply the behavior change wheel of Michie et al. (19), which conceptually illustrates how the components of the COM-B framework that interact with behavior (i.e., capability, opportunity, and motivation) can be affected by one or more practical approaches (i.e., interventions designed to modify one or more components of behavior), which in turn can be influenced by policies (i.e., actions that enable or support interventions). This tool appears to be more accessible to the general practitioner and translatable to the context of animal biosecurity.

There is a distinction (and for those not trained in behavioral sciences, some confusion) among what is “done to” someone as the defined intervention, the behavior change technique(s) being implemented, and the mechanism(s) of action induced “within” someone to lead to (or away from) enacting a behavior. Behavioral scientists are developing taxonomies and catalogs of techniques to help people to implement evidence-based intervention and implementation programs (24). This may be useful in some contexts, but it is unlikely that the evidence base will account for every type of behavior in every context. Those interested in solving practical problems rather than validating theories can also benefit from integrating evidence-based theory-driven elements (25). Even if taking a more empirical approach, by evaluating the successful achievement of the desired behavior as well as a positive outcome of that behavior that leads to positive feedback, effective ways to successfully motivate and sustain behavior can be identified. A single program or intervention may contain multiple components, and methods should be reported in enough detail to clarify how they relate to one another, for instance by using a reporting framework like the Template for Intervention Description and Replication (TIDieR) (26).

The meaning of the term “intervention” varies with context. It can be applied to the specific approach or action used to influence an antecedent of behavior change as well as to the program to deliver behavior change techniques or methods. Different levels of intervention are also possible, ranging from a focus on individual animal caretakers to a production system or to government regulatory policies (21). Intervention mapping (21, 25) is a practical approach for designing and evaluating a program of work intended to lead to behavior change in the target audience. Several intervention mapping frameworks exist and are frequently applied in planning human health intervention programs. For example, the comprehensive 10-step intervention mapping framework of Abraham and Denford (27) extends to scaling up and further evaluation of process and effectiveness, after initial implementation and evaluation of effectiveness. Alternatively, the six-step intervention mapping framework of Bartholomew and others described by Kok et al. (21) starts by (i) developing a logic model of the problem detailing the behaviors, environmental conditions, and key factors contributing to the problem followed by (ii) creating a logic model of change that underlies (iii) program design, realized in (iv) program production (including pre-testing) and (v) program implementation. The sixth step, (vi) evaluation, confirms whether the objectives have been achieved (20). A logic model commonly presents the components of a program in a series of causal, “if-then,” statements connecting resources, activities, and outcomes (28). Here it also connects the proximal change objectives or determinants of behavior with behavioral and environmental changes or outcome objectives (21). The intervention mapping framework also can be used as a lens through which to evaluate efforts to influence behavior in any context. For example, Glanville et al. (29) applied this framework to evaluate interventions targeting animal care behaviors and presented a modified framework. Evaluating studies through an intervention mapping lens is challenging when intervention mapping was not applied in their development but may shed light on the benefits of doing so.

The overall goal of this scoping review was to provide insights into previous work and future efforts to motivate people who work with livestock (for example, farmers, ranchers, producers, and other livestock care takers) to implement biosecurity measures to protect agricultural animal health at the farm or production level. We aimed to encourage the use of more theoretically grounded and potentially effective methods when designing and implementing interventions to improve biosecurity in animal agriculture.

## 2 Methods

This scoping review adhered to the Preferred Reporting Items for Systematic reviews and Meta-Analyses extension for Scoping Reviews, PRISMA-ScR, guidelines (30). As a scoping review, the aim was to summarize the characteristics of studies meeting the eligibility criteria rather than assess effect sizes or conduct meta-analysis. We registered the protocol for this review with Open Science Framework (OSF)^1^ on 19 November 2024, detailing our search of protocol registries for similar efforts. No protocols appeared to overlap with our objectives. Our first objective was to determine what types of interventions have been tested in relation to the uptake of behaviors related to animal disease biosecurity at the farm or production level. By applying the intervention mapping framework of Kok et al. (20) as our evaluation lens, we identified intervention elements, including those describing behavior change mechanisms and clearly linked methods. Because we hypothesized that very few of this type of intervention study had been done, our secondary objective was to determine how methods of a particular intervention map to well-recognized mechanisms of behavior change articulated by behavior change models or theories, regardless of the study’s stated theoretical framework. Specifically, we mapped interventions to components of the behavior change wheel and COM-B framework (19).

### 2.1 Search strategy

We selected a range of databases to identify studies from multiple disciplines including veterinary science, animal science, social science, extension/outreach, and communication. Using the search strategy and terms shown in Table 1, we queried the following databases: CAB Abstracts, Communications and Mass Media complete, Education Resources Information Center (ERIC), MEDLINE, SCOPUS, and Web of Science Core Collection. The initial searches were conducted on 25 November 2024 and repeated on 14 May 2025 to capture records added to the databases in the interim. All searches were completed at Charles Sturt University, Wagga Wagga, Australia.

**Table 1:** Databases and search terms used for this scoping review of published research about the effectiveness of interventions intended to improve implementation of, or compliance with, on farm biosecurity measures.

Database	Time frame	Search options	Search terms^1^
CAB abstracts (via OVID)	1910–2025, week 19	Titles only In English	“bio?security measure” or “bio?security routine” or “bio?security compliance” or “on farm bio?security” or “farm bio?security”
Communications and Mass Media complete (via EBSCOhost)	2008–2023	Entire documents	“biosecurity” or “bio-security”
ERIC (Education Resources Information Center; via EBSCOhost)	2007–April 2025	Abstracts In English Peer-reviewed Journal articles Academic journals	“biosecurity” or “bio-security”
MEDLINE	1996–2025, week 19	Titles only	“bio?security” and “farm*3 or ranch*3”
SCOPUS	Complete since 1996, some journals since 1970; last queried 14 May 2025	Titles only	{biosecurity measure} OR {biosecurity routine} OR {biosecurity compliance} OR “on farm biosecurity” OR “farm biosecurity”
Web of Science Core Collection	1900–present, updated 10 May 2025	Titles only	“biosecurity measure” OR “biosecurity routine” OR “biosecurity compliance” OR “on farm biosecurity” OR farm biosecurity

1 The search character “?” allows for words with or without a character or letter; the search code “*3” looks for the preceding word within three words of the first search term.

Records retrieved from each database were uploaded into Covidence (Melbourne, Australia). De-duplication was initially undertaken in Covidence, followed by additional removal of duplicates using Endnote 21 (Clarivate; London, United Kingdom) and manual checking.

In this review, we refer to “record” as any bibliographic citation captured during database searches and “study” as an individual experiment or initiative. The relationship of “records” to “studies” may not always be one-to-one.

### 2.2 Criteria for inclusion

To be eligible, studies had to be peer-reviewed primary sources in English, have full text available, and report an investigation in which the objective was to evaluate one or more behavior change interventions aimed at protecting animal health from infectious disease challenges at the farm level. Our criteria included studies of biosecurity in the context of any agricultural or farmed animal, terrestrial or aquatic, where the outcome of interest following an intervention was the implementation of one or more biosecurity measures requiring behavior change or facility improvement at the farm level. Studies conducted in the context of any contagious disease, including parasitic infestations, were eligible for inclusion, although a specific disease context was not required. Studies of biosecurity following a disease outbreak were excluded where the outbreak itself or regulatory responses could be considered the intervention. Our focus was on efforts that were intended to increase the ongoing adoption of biosecurity measures. All publication dates indexed within the databases when searched were eligible.

### 2.3 Screening and data extraction process

The review protocol included three levels: Level 1, screening on title and abstract; Level 2, screening on full record; and Level 3, data extraction. The screening rubrics are provided in Supplementary Tables 1, 2. To ensure clarity of the eligibility criteria and rubrics, a subset of records at each level was independently reviewed by all team members. The team subsequently met to discuss discrepancies, clarify criteria, and make adjustments to the rubric where necessary.

At each screening level, two authors independently screened each record—the first author and a co-author who was responsible for a subset of records. No artificial intelligence (AI) tools were used during screening. The web-based review platform Covidence was used to facilitate and track screening decisions at Levels 1 and 2. Discordant recommendations were resolved via meetings with the first author and at least one other team member. The first author and other co-authors performed data extraction independently in parallel, such that data from each record were extracted by two authors using a spreadsheet (Microsoft Excel; Redmond, Washington, USA). Discrepancies were resolved through discussion. Where a single record reported multiple studies, data were extracted separately for each study.

For data extraction (Level 3), we created an extraction framework based on PICO (population/participants, intervention, comparison, outcome), modified to suit our objectives (31) and facilitate analysis through the lens of intervention design by adding identification of context and theoretical framework. We identified the context of the study (i.e., where and why), the human subjects targeted by the study (i.e., who experienced the intervention), the outcome indicators (i.e., what knowledge, attitudes, skills, or practices would change), the intervention (i.e., what activities were done and when), how the intervention or implementation program was evaluated (i.e., what was the evaluation comparator for the outcome indicators), and the theoretical framework underpinning the work. Alignment of the extracted data with the six intervention mapping steps of Bartholomew Eldredge cited by Kok et al. (20, 21) is summarized in Table 2.

**Table 2:** Relationship between data extraction plan and intervention mapping^1^ for this scoping review of published research about the effectiveness of interventions intended to improve implementation of, or compliance with, on farm biosecurity measures.

Intervention mapping step	Data extracted	Location of results
1: Understanding of the problem and its determinants	Study context (i.e., location, livestock species, disease focus, target participants)	Figure 3, Tables 5–7, Supplementary Table 3
2: Determining what needs to change	Outcome indicators and change targets	Supplementary Tables 4, 5
3: Choosing what behavior change methods to use	Theoretical basis of methods	Supplementary Table 4
4: Designing the program and program materials	Intervention activities and components	Supplementary Tables 4 and 5
5: Implementing the program	Length, timing, and cadence of activities	Supplementary Table 4
6: Evaluating the program	Evaluation comparator	Supplementary Table 4

Based on intervention mapping framework of Bartholomew and Eldredge as cited by (20) and (25).

### 2.4 Search validation and adjustments

Prior to data extraction at Level 3, database searches were repeated and reference lists of all records selected for extraction were screened manually for potentially eligible records missed by the original search strategy (32); any found manually entered screening at Level 2.

### 2.5 Data analysis

Data cleaning and descriptive analysis were performed using Microsoft Excel (Microsoft 365 Apps for Enterprise Version 2602). Interventions were categorized inductively based on study descriptions (e.g., audits, interviews, or surveys). Intervention format (i.e., in-person, online, one-on-one, or group) was noted. Outcome indicators were similarly categorized inductively based on study descriptions (e.g., attitudes, beliefs [or risk perceptions], health outcomes, knowledge, practices or behaviors, skills, or other). We did not evaluate how or to what extent theoretical frameworks were integrated into the reported studies, nor did we formally assess the rigor of the study designs employed.

### 2.6 Mapping to the behavior change wheel and COM-B framework

**Figure 1:**
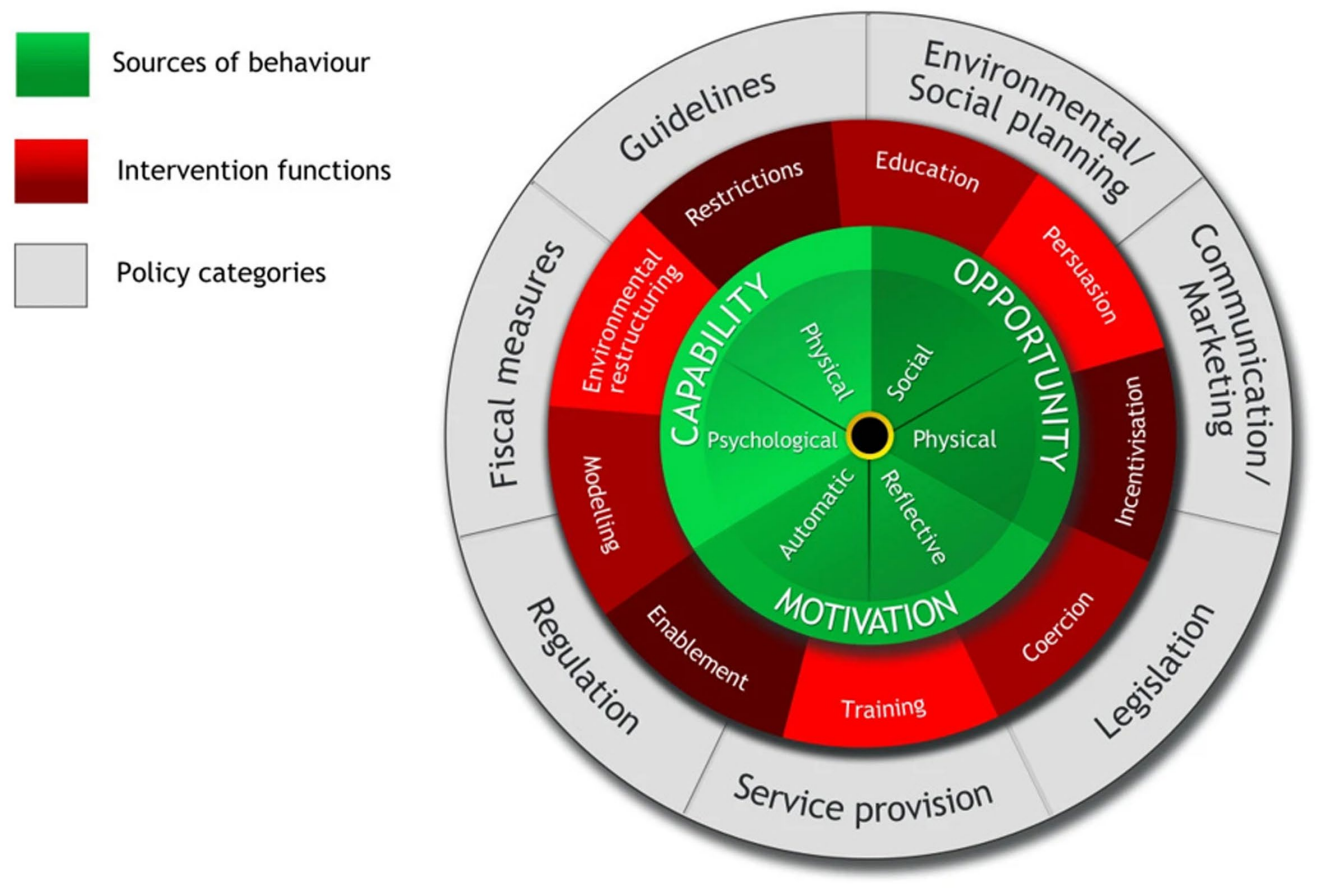
The behavior change wheel of Michie et al. (19). Reprinted under Creative Commons Attribution License (http://creativecommons.org/licenses/by/2.0).

We mapped interventions to the elements of the behavior change wheel (19), an accessible presentation of categories of ways to influence behavior change, while recognizing that these categories are evolving within health behavioral science and other disciplines. The initial categories of policies and intervention functions followed Michie et al. (19), the policies being communication/marketing, environmental/social planning, fiscal measures, guidelines, legislation, regulation, and service provision; and the intervention functions being coercion, education, enablement, environmental restructuring, incentivization, modeling, persuasion, restrictions, and training (Figure 1). Two authors independently mapped each study—the first author and a co-author who was responsible for a subset of records. After independently mapping the studies, the authors met to discuss their choices and justifications and arrived at a final consensus mapping. We considered creating new categories when existing ones were not a good match. A similar process was used to map intervention components to sources of behavior according to the COM-B framework. The behavioral antecedents (C, O, and M) were not usually stated in the study descriptions, so the mapping was done inductively based on the descriptions of the intervention components. Again, discrepancies between the two authors who mapped each study were resolved through discussion.

In the process of mapping interventions to the behavior change wheel, we proposed creating a new policy category called information gathering/elicitation, which we defined as using surveys, interviews, or observation to collect data. Correlated with the proposed policy category, we also proposed an intervention function of questioning/observing, defined as collecting information about the individual (i.e., knowledge, attitudes, beliefs, or perceptions) or about biosecurity measures in place. These types of interactions with individuals responsible for enacting biosecurity measures were posited to have inherent impacts on automatic motivation regardless of their stated purposes. Items mapped to the policy of information gathering/elicitation and the related intervention function of questioning/observing are shown in Supplementary Table 5. We also considered adding an intervention function of planning but realized that would conflate a delivery method with a cognitive activity (33). Therefore, we mapped planning activities (e.g., involvement in developing or committing to a plan of action) to the policies of guidelines, regulation, or service provision and to the intervention of persuasion, although an argument could be made for connecting it with enablement as well. Cues to action such as coaching, commitment, incentives, and monitoring were also mapped to persuasion. The final set of policy categories and intervention functions, showing our additions and examples relevant to biosecurity contexts are shown in Tables 3, 4. These tables illustrate how common activities used in biosecurity programs—such as surveys, coaching, planning, monitoring, and advisory visits—align with components influencing behavior change.

**Table 3:** Extension of behavior change wheel policy^1^ categories to biosecurity contexts.

Policy category	Definition	Biosecurity example
*Information gathering/elicitation*	*Using surveys, interviews, observation to collect data*	*Attitude (e.g., ADKAR) or biosecurity assessments (e.g., BEAT, BioCheck, Secure Food Supply)*
Communications/marketing	Using print, electronic, telephonic, broadcast media, (*or in-person delivery*)	*Websites, workshops, media campaigns, etc*.
Guidelines	Creating documents that recommend practice (*i.e., voluntary*)	*Plans, protocols, checklists, etc*.
Fiscal	Using the tax system or contracts to reduce or increase financial cost	*Aligning payment terms with biosecurity implementation or health outcomes*
Regulation	Establishing rules or principles of behavior or practice (*i.e., creating industry or market-driven mandates*)	*Quality assurance programs, international (WOAH) animal health codes*
Legislation	Making or changing laws (*i.e., creating legal mandates*)	*National laws regarding animal biosecurity*
Environmental/social planning	Designing and/or controlling the physical or social environment	*Biosecurity measures at the level of the physical facility (e.g., facility design, production network policies, entranceway design, posting of signs, locking gates, etc.)*
Service provision	Delivering a service	*Veterinary or technical/extension services to support implementation of preventive practices including plan development, commitment, and monitoring; also efforts to enhance capacity to support general animal health and well-being*

1 Here policy refers to a means of providing interventions.

2 Adapted with modification from Michie et al. (2011), under Creative Commons CC by 2.0 (https://creativecommons.org/licenses/by/2.0/).

The italicized elements represent adaptations of the framework^2^ to reflect practices commonly used in livestock health and biosecurity contexts.

**Table 4:** Extension of behavior change wheel intervention function^1^ categories to biosecurity contexts.

Function category	Definition	Biosecurity example
*Questioning/observing*	*Questioning or observation to compare practice with recommended best practice*	*Utilize surveys, checklists, assessments, or audits (video, in-person, or self-assessment)*
Education	Increasing knowledge or understanding	*Provide preventive health information or advice*
Persuasion	Using communication to induce positive or negative feelings or stimulate action	*Add content designed to generate an affective response when providing information or advice*; *co-create a preventive health plan or Secure Food Supply plan*; *coach, prompt, or monitor implementation of plan*
Incentivisation (*reinforcement*)	Creating expectation of reward	*Expect better animal health if enact new practice(s); provide bonuses for meeting desired score or implementing specific practices*
Coercion (*punishment*)	Creating expectation of punishment or cost	*Anticipate greater likelihood of animal disease if do not enact new practice(s); assessment of penalties (or loss of market access) for failing to meet desired score or enact a specific practice*
Training	Imparting skills	*Provide clear instructions or demonstration of a skill, possibly in a situation that facilitates its practice*
Restriction	Using rules to increase (or decrease) the opportunity to engage in the target behavior	*Require health papers before interstate/international animal movements*
Environmental restructuring	Changing the physical or social context	*Provide signage, security, drover’s lanes, Danish entries, uniforms, etc*.
Modeling	Providing an example for people to aspire to or imitate	*Apply demonstration farm concept*
Enablement	Increasing means/reducing barriers to increase capability or opportunity	*Provide a resource (e.g., financial, physical, or advisory) to support enactment of new practices*

1 Here intervention refers to what target individuals receive.

2 Adapted with modification from Michie et al. (2011), under Creative Commons CC by 2.0 (https://creativecommons.org/licenses/by/2.0/).

The italicized elements represent adaptations of the framework^2^ to reflect practices commonly used in livestock health and biosecurity contexts.

## 3 Results

**Figure 2:**
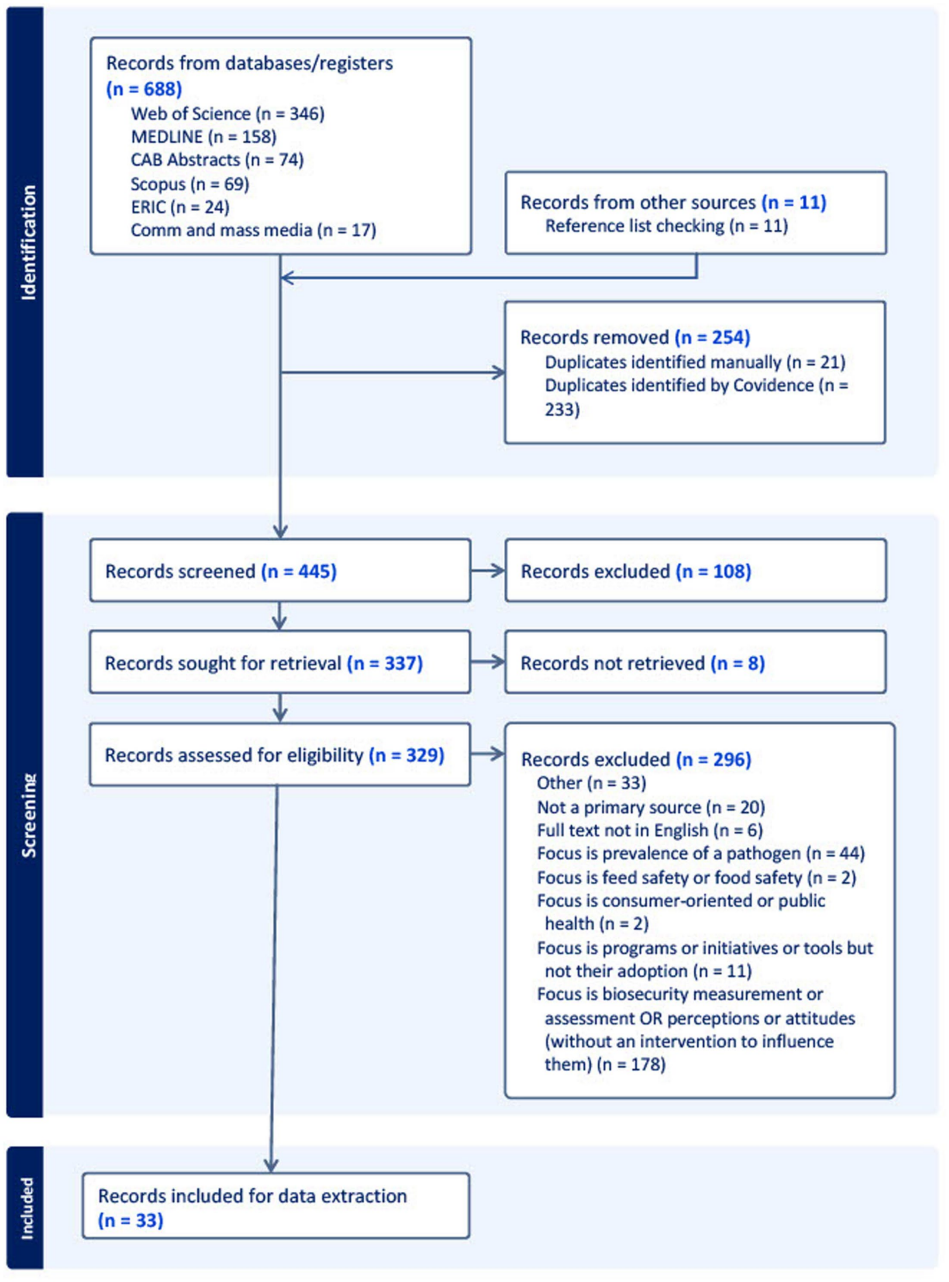
PRISMA flow chart illustrating number of records identified and retained at each stage of the search and screening process.

Of the 699 records imported for screening (688 from database searches and 11 from checking reference lists), 445 remained after de-duplication. Of these, 108 records were excluded at Level 1, and eight did not have full text retrieved. The remaining 329 proceeded to full text screening (Level 2), from which 33 records were eligible for data extraction (Figure 2). In two cases, findings from a single study were reported in two separate papers [(34, 35) and (36, 37)]. In each case, these were counted as a single study for analysis. Seven records reported more than one experiment or evaluation of different populations, and each was considered as a distinct study for analysis. Thus, from these 33 records, 41 distinct studies were identified; hence the numbers of studies presented in the following tables sum to more than the number of records extracted.

Most exclusions (60%) were studies that only measured biosecurity behavior or attitudes and did not report an intervention designed to change behavior. About 15% assessed biosecurity but were primarily focused on the prevalence of one or more pathogens, rather than interventions intended to change biosecurity practices. In addition, nearly 7% (*n* = 20) were excluded because they were not primary sources, and 2% (*n* = 6) were excluded because they were not published in English. A spreadsheet of screened records that were excluded is provided in Supplementary Materials.

The following sections summarize the contexts (i.e., geographic locations, livestock species, and diseases of interest), the types of participants targeted by the interventions, the categories of desired outcomes (i.e., knowledge, attitudes, skills, or practices), the intervention program methods or components (e.g., audits, interviews, or surveys), the stated theoretical frameworks informing the work, and selected elements of evaluation (i.e., the experimental design and outcome indicators) of the studies that were included. Additional details are presented in Supplementary Tables 3, 4.

### 3.1 Study context and participants

**Figure 3:**
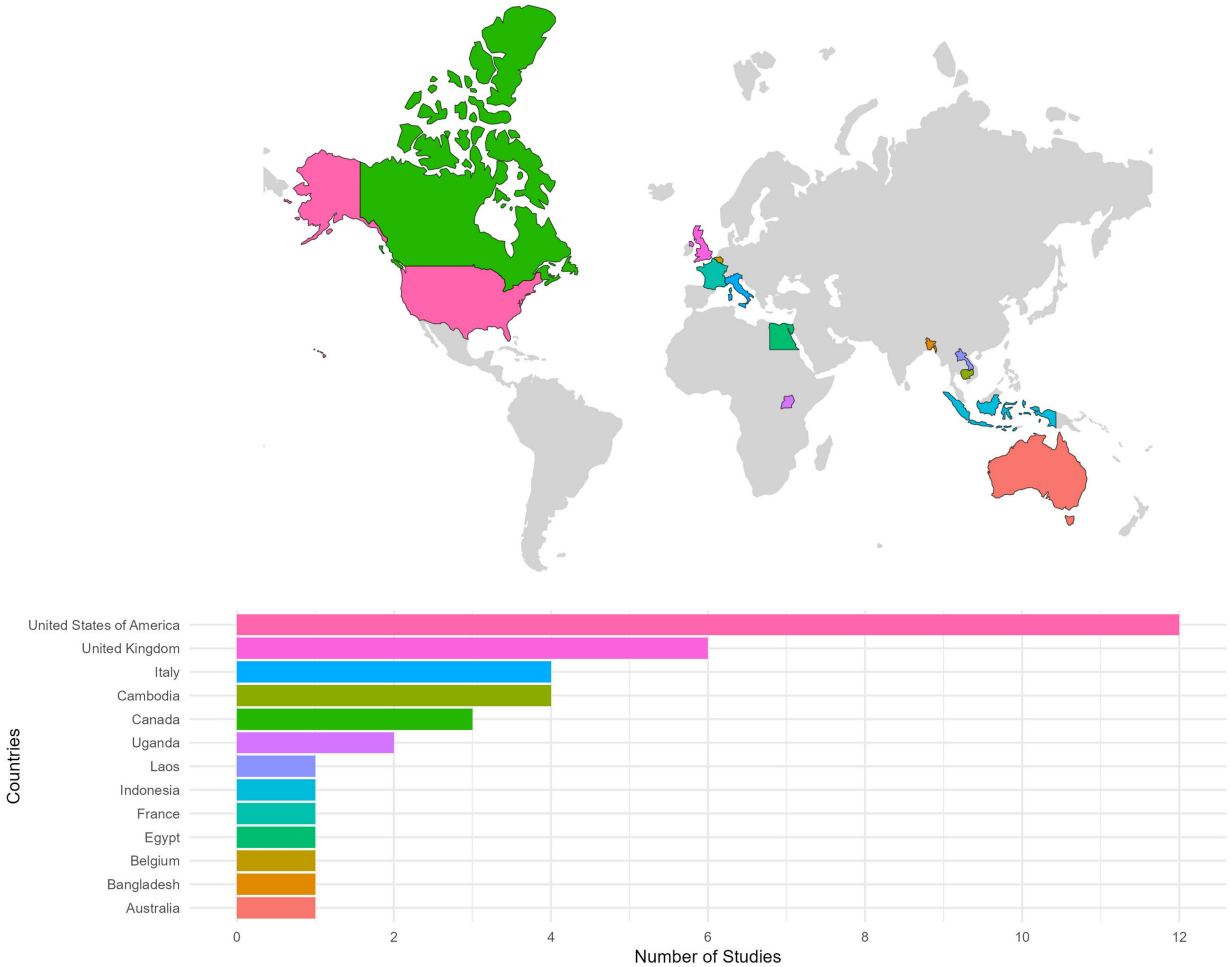
Geographical location of studies extracted in this review of biosecurity implementation studies.

The studies included in this review were conducted on all continents except South America and Antarctica (Figure 3). The greatest number were conducted in the United States of America (U.S.; *n* = 12), the United Kingdom (*n* = 6), and Cambodia (*n* = 4). Studies generally focused on biosecurity in the context of a single type of livestock, most commonly poultry or swine (Table 5), with the exception of the U.S. studies related to 4-H projects, which did not specify a species, and studies involving both cattle and buffalo in southeast Asia. When describing the context of the study, 25 did not specify a particular disease of interest (Table 6), whereas some were concerned with more than one. Several studies were concerned with zoonotic or high consequence diseases like avian influenza, salmonellosis, African swine fever, or foot-and-mouth disease. A few were concerned with endemic diseases like parasitic infestations or footrot. Thirty studies were conducted between 2008 and 2023, while 11 did not specify when they were conducted, although they all were published between 2011 and 2024.

**Table 5:** Livestock species under consideration in studies reported in records extracted for this scoping review of published research about the effectiveness of interventions intended to improve implementation of, or compliance with, on farm biosecurity measures.

Livestock species	No. of studies
Cattle: beef or dairy, or buffalo	6
Poultry: chicken, ducks, or turkeys	14
Sheep	4
Swine	15
General (4-H projects^*^)	2

* 4-H projects are learning endeavors conducted by youth in an area of interest (https://4-h.org/).

**Table 6:** Disease focus of studies in records extracted for this scoping review of published research about the effectiveness of interventions intended to improve implementation of, or compliance with, on farm biosecurity measures.

Target disease	No. of studies*
None specified	25
African swine fever	2
Avian influenza	2
Foot-and-mouth disease	3
Footrot	3
Parasites	2
Salmonella	2
Other infectious disease**	6

*Not mutually exclusive; hence sum is greater than 41.

**Hemorrhagic septicemia, blackleg, bovine respiratory disease, BVD, IBR, *L. hardjo*, MAP, *M. bovis*, or Newcastle disease, on own or in combination with one of the above.

The targets of the reported interventions (shown in Table 7) can be broadly categorized as youth; adults not engaged in animal production; smallholders; and commercial producers, farm workers, or advisors like veterinarians. In most cases, the targets of the reported interventions were also individuals expected to engage in biosecurity-related behaviors. Of the records extracted, 13 studies interacted with targets who were not necessarily regularly engaged in on-farm activities (i.e., 4-H youth, contractors, farm visitors, or others), including Amazon Mechanical Turk, an online platform for recruiting participants (known as Turkers) to complete various tasks.

**Table 7:** Target stakeholder expected to perform biosecurity measure(s) in studies included in this scoping review of published research about the effectiveness of interventions intended to improve implementation of, or compliance with, on farm biosecurity measures.

Target stakeholder(s)	No. of studies	Citation
Youth	1	(53)
Amazon Mechanical Turk*	4	(42–45)
Contractors	1	(39)
Farm visitors	3	(2 studies in 54, 1 in 55)
Other adults not engaged in farming	4	(2 studies in 43, 1 each in 47, 53)
Commercial producer, farm worker or advisor	25	(2 studies in 54 and 50, 3 in 68, 4 in 46, 1 each in 35, 38, 47–49, 52, 55, 56, 69, 92–96)
Small holders with cattle	3	(2 studies in 40, 1 in 41)
Small holders with pigs	2	(51, 64)
Small holders with poultry	3	(37, 56, 78)

*Amazon Mechanical Turk is an online platform for recruiting participants (known as Turkers) to complete various tasks (https://www.mturk.com/).

### 3.2 Outcome indicators

The outcome indicators of each intervention, in terms of what was measured and how the indicators were categorized, are presented in Supplementary Table 4. These varied widely, from web traffic metrics and short-term knowledge gains to changes in attitudes and behaviors. Most studies had a stated or implicit goal of increasing knowledge, typically through educational interventions; but only 15 studies directly measured knowledge outcomes. Many studies aimed to generate a behavior (*n* = 31), i.e., an observable action, but not all measured behaviors were directly related to biosecurity practices. A few studies were focused on skill development (*n* = 3), although these studies did not always assess whether newly acquired skills were translated into practice. Other studies were designed to facilitate a change in attitudes or beliefs (*n* = 10). In addition, some studies (*n* = 11) included a measure of a health outcome or biomarker of some kind. One study measured intentions relative to specific biosecurity measures (38), while another focused primarily on policy outcomes (39).

### 3.3 Intervention length and activity

Key elements of interventions are length (or intensity) and intervention activity (or type of interaction; columns two and three of Supplementary Table 4). Studies varied from one-time interactions to repeated visits at different intervals of time. Intervention activities included assessments of knowledge, attitudes, and practices; risk assessments; audits and monitoring; educational delivery, advice, and action plans; information or message treatments; model farms; and contract analysis. Seventeen relied on education as the intervention approach (Supplementary Table 5). Further analysis of the interventions is discussed in the intervention components section below.

### 3.4 Evaluation

The evaluation comparators (column four of Supplementary Table 4) were driven by the experimental design used in each study. Most studies utilized quasi-experimental designs, typically involving within subject or within farm comparisons, with baseline and endline measurements and occasionally intermediate data points. Four studies [(37, 40), and two in (41)] used cluster-randomized designs, and five [(42–45), and two in (43)] used randomized complete or incomplete block designs in experimental simulations of production environments (42–45).

### 3.5 Theoretical grounding

A variety of theoretical frameworks or theories were referenced by the studies included in this review (column six of Supplementary Table 4). Among these theories were the health belief model (46), theory of planned behavior (47), theory of reasoned action (48), pathway to disease control model (38), various theories from behavioral economics (42–45), Hofstede’s cultural dimensions theory (42), the knowledge-attitude-practice model (40), ADKAR (awareness, desire, knowledge, ability, reinforcement) (49, 50), and item response theory (51). One study used an ethnographic approach (52). However, 16 studies did not explicitly state a theoretical framework, although elements consistent with behavioral change theory were often described.

### 3.6 Intervention components

Prior to being mapped to elements of the behavior change wheel, interventions were further characterized by format and components, categorized inductively based on study descriptions (Supplementary Table 5). The intervention component descriptions summarize what the target stakeholder received or responded to, whereas the intervention format describes how the component was delivered. Types of formats included printed, digital, and verbal communications delivered by mail (*n* = 2), online (*n* = 3), or in-person (one-on-one or group-based; *n* = 24), respectively. Environmental manipulation was used in 10 studies, including monitoring technology (54, 55), information scenarios in computer simulations (42–45), and model farms—one study with active engagement of the community (56) and one relying on passive knowledge diffusion (38). One study (52) reported on a 4-H project, which consisted of a series of group, independent learning, and knowledge application activities. Intervention programs frequently involved multiple components such as training, audits of practices, advice or planning, and surveys of attitudes.

### 3.7 Mapping to the behavior change wheel

The mapping of intervention components to elements of the behavior change wheel (19) also can be found in Supplementary Table 5. Components were linked to all behavior change wheel policy categories except legislation, and to all intervention functions except restriction. The intervention functions of education, training, or persuasion mapped to components of 40 studies [the exception being Komaladara et al. (39)]; questioning/observing mapped to 25 studies; enablement mapped to 14 studies; environmental restructuring mapped to 10 studies; planning mapped to seven studies, and incentivization mapped to six studies.

## 4 Discussion

The primary objective of this scoping review was to identify and assess published research about the effectiveness of interventions intended to improve the implementation of, or compliance with, biosecurity measures at the farm level to protect livestock health. To achieve this, we identified and classified efforts where the intention was to make a change in behavior relevant to disease prevention (i.e., to implement one or more biosecurity measures). We then explored theoretical and practical frameworks to support future interventions aiming for successful adoption and routine use of biosecurity measures. Our intention was to identify gaps and limitations in the existing literature and suggest directions for future research. This review identified several weaknesses in how interventions to stimulate the adoption of biosecurity measures in animal agriculture have been conducted and reported, as well as a general lack of transparency regarding the theoretical underpinnings of the work, if they were considered in the first place. We discuss our findings in relation to the intervention mapping framework before concluding with some recommendations for future biosecurity implementation efforts.

### 4.1 Focused on interventions

We focused on characterizing interventions designed to make changes to biosecurity measures in practice rather than assessing either existing biosecurity measures or the psycho-social determinants of making changes. Similar to Glanville et al. (29), we observed (and excluded), a relatively large number of studies that examined attitudes and other antecedents to behavior, while identifying substantially fewer studies that reported designing and testing an intervention. Although identification of attitudes and other antecedents to behavior is important when designing an intervention [as reviewed by Buchan et al. (8)], careful attention is needed to understand which factors apply to the specific behavior (i.e., adoption of the specific biosecurity measure of interest) and focus on these. For instance, implementing hand and footwear hygiene practices is very different from isolating newly introduced animals separately from other animals, and these behaviors are likely to require a different set of approaches to achieve. In fact, Richens et al. (18) found greater association of behavioral beliefs or attitudes with behaviors to prevent indirect transmission, whereas control beliefs were more strongly associated with behaviors to prevent direct transmission of disease. In the studies we reviewed, however, details explaining how specific activities were designed with techniques expected to deliver behavior change mechanisms were typically lacking. An underlying concern is the lack of empirical evidence to support many biosecurity recommendations, but that was not the subject of this review.

### 4.2 Contextualizing biosecurity

Location, participants, disease and species of interest are important aspects of the biosecurity context, but perhaps not sufficient. Although the country in which the study took place may be seen as a proxy for the economic situation of those engaged in rearing animals, this may not always be correct at the individual level. Whether the country is engaged in significant international trade of animals and animal products or is prevented from such trade by endemic transboundary animal diseases may be a more important factor to consider. In this review, most studies were conducted in countries for which international trade is important: Australia, Europe, and North America. We did not extract details reflecting the regulatory and political environment or the organization of animal health services, even where such details were mentioned. Nevertheless, we are aware that important regulatory differences exist, even among higher-income countries. For example, biosecurity has been a feature of the European Union Animal Health Strategy since 2007 and was most recently consolidated within the Animal Health Law of 2016, which came into effect in 2021,^2^ although the implementation of regulations still varies between countries (57, 58). Animal health laws in the U.S., on the other hand, have not directly mandated biosecurity at the farm level, although cooperative industry-regulatory programs exist for poultry (the National Poultry Improvement Program)^3^ and more recently for swine (the U.S. Swine Health Improvement Program).^4^ Regulatory as well as economic and trade contexts should be specified.

Many biosecurity measures offer protection against multiple diseases, which may explain why many studies did not specify a disease context. Although it may be seen as an advantage to get broad protection from a set of biosecurity measures, this lack of specificity makes it difficult to determine the effectiveness of implementing one or more practices, or the contribution of a particular practice to the level of protection achieved. Benefits to productivity could motivate the adoption of biosecurity (59), but the effectiveness and perceived value of specific practices could depend on farm-specific factors. Teasing these out is important because poorly designed studies may contribute to farmer unwillingness to adopt specific practices. Marier et al. (38), for example, tested whether demonstrating a practice, whether effective or not, would influence the beliefs of other farmers in the social network of the demonstration farms. In that study, four different practices to protect against *Salmonella* were trialed on one farm each, but none of the participating farmers believed that any meaningful improvement had resulted. This appeared to reinforce the broader perception that current recommendations were not worth the effort required for implementation. To get good voluntary uptake, the farmers would have had to be sure the measure would be effective and relatively easy to implement (38). It is unclear what effect coercion in the form of legislation or market mandates would have, given the overall low belief in self-efficacy among these farmers.

### 4.3 Alternative intervention approaches

Several studies documented choices taken in response to information treatments presented in multiple rounds of a simulated (online) production environment (42–45). The latter approach was pioneered by the Social Ecological Gaming and Simulation laboratory at the University of Vermont as a way to demonstrate choices related to biosecurity (albeit choices made in an online simulation) in response to information about disease risk, disease and biosecurity status of surrounding farms, certainty of risk message, type and language of message, and role models in the same workplace (60). Such studies necessitate a simulated environment since creating the choice conditions in the real world (i.e., determining how people would respond to cues during an emerging or foreign animal disease outbreak) would be impossible (and unethical). Combining findings from discrete choice experiments with simulation modeling is another approach for evaluating the potential ability of incentives to influence biosecurity actions (61). Studies where the “intervention” was the occurrence of a high consequence or regulatory disease were excluded from this review. Nevertheless, it has been observed that producers can be highly motivated to change after such events (62).

The limits of education interventions in motivating the adoption of certain types of behavior have been well documented (63). Based on producer feedback and research observations, economic barriers are seen as a major hindrance to biosecurity measures requiring a significant investment (40, 64). Regardless of whether the economic barrier is a primary limitation or a rationalization for not implementing biosecurity, it cannot be overcome by education alone. Yet, demonstrating the cost–benefit of most biosecurity measures remains problematic. In cases where disease pressure is significant—for instance, porcine reproductive and respiratory syndrome (PRRS) in the U.S. swine industry—it is easier to demonstrate the benefit of costly investments like air filtration (65, 66). In the absence of consistent disease pressure, or where the concern is an unlikely, but high consequence disease, the potential for linking biosecurity measures to market incentives is a possible option. One study in this review (39) analyzed existing contracts in the poultry industry in Indonesia to identify those most likely to serve as an incentive for investing in biosecurity. The study, however, did not directly assess the biosecurity status of farms under these contracts. Without detailed data on compliance with biosecurity measures as well as animal productivity and health outcomes, it is not possible to make a clear case for the cost–benefit of general improvements in biosecurity. Taking a systems approach to developing appropriate economic incentives in conjunction with cooperative regulatory and industry initiatives may yield better uptake of biosecurity practices (67). In the studies included in this review, we found a greater emphasis on capacity building at the community level in participatory projects conducted in low- and middle-income countries [e.g., (36, 40, 41, 51)] than elsewhere. Participatory projects elsewhere may not be reported in peer-reviewed publications, hence not finding them may reflect a literature bias rather than an actual absence.

### 4.4 Evaluation of interventions

Tracking of both behavioral outcomes and the reasons for not implementing changes is needed to reveal barriers related to the opportunity component of COM-B. Structural and social barriers to biosecurity implementation are commonly noted in studies conducted in low- and middle-income countries, but their influence on decision-making in all contexts could be more rigorously explored. Almost all studies included in this review addressed psychological capability through an educational component, but relatively few evaluated the effectiveness of this component by measuring knowledge gains and the influence of knowledge on behavior. One set of studies tested educational methods to demonstrate (as predicted) that personal interaction was most effective (68). The methods and time frames of evaluating the outcomes of each intervention varied. Some educational interventions assessed only immediate or retrospective pre-post knowledge gains (47, 69), while others were unable to demonstrate measurable changes in knowledge (46). The ease, inexpensiveness, and better response rates associated with documenting short-term knowledge gains contribute to the popularity of this evaluation strategy, although it is less rigorous than comparing true baseline to endline measures as done by MacPhillamy et al. (40). Several interventions included repeated follow-up visits or surveys. However, it is important to recognize that formative and summative assessments may themselves function as interventions. The “question behavior” effect has been documented in psychological research (70). Its role in motivating behavior when interventions include surveys, interviews, and other types of assessment is unknown but can be suspected to play a role. As noted by Ellis-Iverson et al. (71), tactics to encourage sustainable behavior change such as monitoring, benchmarking, and audits have not been adequately evaluated in terms of their influence on sustained compliance and warrant further investigation. What may be seen as the evaluation program could also be acting as a motivational tactic. Included in this scoping review were both one-off studies and studies involving several years of interaction with a community. Although time to final evaluation is usually limited by funding constraints, the appropriate time interval over which to assess lasting effectiveness of a particular intervention, even if unattainable, should receive more consideration.

We identified a common weakness in study designs of not incorporating control groups, so the evidence base is largely quasi-experimental. Most studies utilized a quasi-experimental within-subject or within-farm design, which allowed measurement of change following the intervention, but did not include a true control group. In studies where the intervention included coaching or other touch points between audits, it would be impossible to separate the effect of the repeated audits from the other parts of the intervention without a non-contact control group. Cardwell et al. (72), for example, compared the effect of providing tailored versus generic advice on the subsequent years’ biosecurity assessment scores and demonstrated improved scores regardless of treatment. This is supportive of the question-behavior effect mentioned above, but not conclusive as there was no group that did not receive advice. The most rigorous study designs among studies in this review were cluster randomized trials, assigning villages to intervention or “control” treatments (which were usually a different level of intervention) as in Conan et al. (37), MacPhillamy et al. (40), and Nampanya et al. (41). Randomized controlled designs were also utilized in the experiments conducted via online simulations (42–45). The ease of conducting randomized controlled trials in simulated situations is advantageous. If decision-making of the sample “tested” is similar to decision-making among farmers (73), such studies also circumvent higher rates of survey refusal reported among U.S. farmers (74). Another way to accelerate the identification of interventions with the most impact and least effort given limited resources is to implement the one group pretest-posttest design in different areas of the same country simultaneously but with different interventions. In the context of forest biosecurity (which was not within the scope of this review), Aley et al. (75) used this approach to test five different interventions to facilitate compliance with measures to prevent spread of invasive species by users of hiking trails. Coupling such intervention testing with cost–benefit analyses would further assist in identifying the most promising interventions. Future studies should therefore be designed to identify the effects of specific elements expected to improve motivation, such as co-creating the plan, committing to the plan, receiving coaching or other prompts versus annual re-assessments or audits themselves. The field would also benefit from an evidence base that could guide decisions about when education, audits, or enablement are most appropriate to achieve improvements in biosecurity.

### 4.5 Use of theory-based approaches

In the process of data extraction, it was not our intent (or even possible) to determine the authors’ philosophies in relation to using theoretical approaches. Some authors may not use them because they are simply unfamiliar with behavior change theories and their application; other authors may gravitate towards theories that are simple or popular. The vast number of theories that exist can be intimidating (76). When it comes to developing interventions to change behavior, it is helpful to recognize that because behavior is influenced by many factors, behavior change strategies can draw on several theories simultaneously (25, 77). In support of using a theoretically grounded approach, Green et al. (77) recommend, “To advance knowledge about effective behavior change strategies, further studies should be done to clearly demonstrate how the intervention design is informed by the theories, and to measure the theoretical constructs before and after the intervention, as well as the associated changes in behavior.” This assumes a construct or determinant is adequately targeted, assessed, and actually correlated with the behavior change. As an alternative or in addition, qualitative methods such as ethnographic approaches [for instance, as used by (78)] can provide useful information by documenting patterns of social interaction within the local context and the perspectives of participants in their own words. Chamberlain and Lyons (79) further challenge the dominance of positivist thinking in the field of behavior change and call for broadening multi-disciplinary inclusion and consideration of social-ecological contexts. A further critique of traditional mathematical approaches comes from Heino et al. (80), who suggest that it could be more reasonable to think of behavior as happening in a dynamically adjusting context, requiring complex systems approaches to model and analyze behavior. Study designers therefore need to identify which conceptual pathway is most appropriate for their aims and context.

Both the KAP approach and Knowledge-Skills-Intentions program models tend to downplay the role of external factors, namely the “opportunity” of the COM-B framework, including social influences as well as economic and other resources. Other behavior change frameworks such as social marketing of behavior (81–83) and the nine dimensions of influence encapsulated by the acronym MINDSPACE (84, 85), although not as comprehensive or coherent as the theoretical domains framework (19), put more emphasis on social norms and activating automatic motivation towards enacting behavior. They were not mentioned by studies identified in this review, but they could be useful tools for intervention designers who move beyond communication-focused approaches.

Integrating social-ecological models such as the pathway to disease control (71) with an intervention mapping framework [e.g., (20, 21)] seems a natural next step for planning interventions targeting the implementation of biosecurity measures. This type of social-ecological approach ensures that important determinants of behavior are not overlooked (21). If external factors are limiting (i.e., the opportunity component of COM-B), they must be addressed; otherwise, the success of interventions focused on capability will be limited. It follows that multi-disciplinary and multi-scalar, that is, system-based, approaches are likely to be more successful. For instance, when the target behavior is not just a new behavior but a change of the existing ways of doing things, achieving the new behavior is much harder. In this case, bringing about behavior change at the individual level could be facilitated by interventions aiming to adjust external conditions—economic, social, environmental, or regulatory—to support new ways of doing things (86). As behavior change models are applied to human behaviors intended to protect animal health rather than those that directly affect personal or human health, it is important to recognize that drivers of behavior change may differ in these contexts.

**Figure 4:**
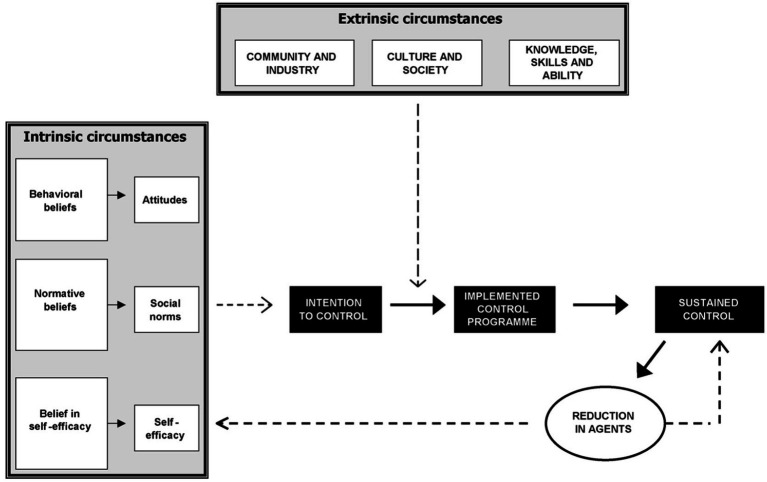
The “pathway to disease control” model of Ellis-Iversen et al. (71), which is a modification of a model originally proposed by Panter-Brick et al. (87). Reprinted from Ellis-Iversen et al. (71) with permission from Elsevier.

Among the behavior change models explicitly integrated into studies in this review, only one was based on a model developed specifically for farm settings. Marier et al. (38) utilized the “pathway to disease control” model (71, 87), an extension of the theory of planned behavior (14). Ellis-Iversen’s model includes external factors (identified as extrinsic circumstances), which reflect the farmer’s position as a businessperson not just concerned with animal health but also affected by market forces and customer demands, industry norms, and availability of technical and financial support (Figure 4). By incorporating four stages relative to taking action from no intent to sustained effort, this model also incorporates some elements of the stages of change or trans-theoretical model (88), clarifying the need for different approaches to generate intention, implementation, and maintenance of the behavior. By positioning the decision frame between intrinsic and extrinsic factors, the “pathway to disease control” is a type of social-ecological model because it includes factors at scales beyond the individual level. Moving out from the personal level, interpersonal and contextual/environmental factors can be considered (21). These levels of influence are also reflected in intervention mapping (20, 21), the COM-B framework (19), and the behavior change wheel (19).

### 4.6 Mapping to existing behavior change frameworks

Mapping intervention components to behavior change wheel (19) categories in this review proved challenging because the study descriptions did not necessarily provide enough detail and did not clearly fit some descriptions of categories that were developed in human medical contexts. The behavior change wheel is intended to support the design of effective interventions and, with the modifications we proposed, it could be more practical for those working in livestock biosecurity contexts. We proposed a new policy category of information gathering/elicitation and a related intervention function of questioning/observing. These additions simplify the application of the framework to animal health contexts and highlight the impact of focusing attention on areas of management. The routine collection of information about livestock caregivers’ beliefs, attitudes, and practices and the health status of the animals under their care is commonplace, regardless of whether it is part of a formal research program or an integral aspect of veterinary practice, and should be recognized for its role in influencing automatic or reflective motivation. Examples relevant to biosecurity contexts in Tables 3, 4 are meant to be illustrative, not limiting.

Unlike Dogusan et al. (9), we did not attempt to connect interventions with specific domains or underlying constructs of the theoretical domains framework (23). However, we similarly found that few studies employed tactics expected to elicit automatic motivation. Motivation, either automatic or reflective, refers to the process that underlies goal-oriented behaviors (19). The abundance of knowledge-attitude-practice (KAP) logic models that emphasize psychological capability and reflective motivation as precursors of behavior may be missing an important target, when, as Michie et al. (19) pointed out, reflective motivation is the only element of the COM-B framework that is *not* absolutely essential to generate behavior.

### 4.7 Recommendations

Developing and delivering effective interventions to motivate the adoption of biosecurity measures requires careful consideration of the socio-psychological and economic drivers of behavior. Based on the common limitations encountered in this review of studies aiming to enhance biosecurity implementation, our recommendations are two-fold: (1) follow an intervention-mapping process for planning the project, and (2) use an appropriate template when reporting on the work for completeness, transparency, and repeatability. More intentional planning and reporting should lead to better clarity around what was done and why, which then informs evaluation of the intervention.

Those utilizing an intervention mapping process will describe the problem and its context; develop a model of change (e.g., logic model or theory of change model); plan, design, and conduct the program to achieve the desired change; then evaluate the outcomes. This is very similar to the approach used in community-based social marketing (83). However, in the social-ecological context in which livestock are managed, those planning interventions need to consider the full range of available tools [i.e., the middle and outer rings of the behavior change wheel (19)], not just beliefs, which are the main change target of social marketing.

One source of guidance and a checklist for describing methods is known as TIDieR (26). The 12-point checklist includes: (1) a brief description or name of the intervention, (2) a rationale for the essential elements of the intervention (e.g., theory or evidence base), (3) a description of materials provided to participants or those being trained (and where they can be accessed), (4) exactly what was done to conduct the intervention, including any enabling or support activities, (5) who provided the intervention and what were their credentials or training, (6) how the intervention was delivered, i.e., face-to-face versus online, individually or in a group setting, (7) where the intervention occurred, (8) dose, interval, and frequency (i.e., number and length of sessions and interval between them), (9) whether interventions were tailored or customized in some way, (10) modifications that occurred to the intervention over the course of the study (if applicable), (11) how intervention fidelity was tracked and assessed, and (12) how well the intervention was delivered. Items 10 and 12 can only be reported retrospectively. Focusing especially on items 2 and 4 in the TIDieR checklist would substantially improve the utility of published studies in informing future efforts. At the very least, it is imperative for researchers to clearly describe: (a) change targets (e.g., knowledge, skills, attitudes, or behaviors); (b) intervention components (e.g., surveys and monitoring, training, or action plans); and (c) delivery formats (e.g., in-person, mail, or online simulation).

Even when following a checklist, authors may neglect to specify which determinants and beliefs were targeted with what methods, the practical application of the methods, and how the parameters for effectiveness, population characteristics, and context were respected (or not) when translating methods into practical application (24). These points are as important in animal health contexts as they are in public health (89), and authors are encouraged to include descriptions of them to the extent possible. In addition, those interested in fostering the application of biosecurity measures will need to consider whether their program is testing an intervention or is applying a tested intervention. The former has an evidence-based rationale (i.e., can be developed by following intervention mapping), whereas the latter incorporates an evidence-based intervention and is, in fact, an implementation program (90).

### 4.8 Limitations of the review approach

We limited our scope to peer-reviewed papers published in English, which likely reduced the geographic representation and range of study types included. Our inclusion of only papers with “biosecurity” in the title or abstract may have inadvertently excluded work that otherwise met the inclusion criteria, such as work being done on mastitis control (91). The process of mapping interventions to elements of the behavior change wheel revealed different thought processes among authors. While we used discussion to reach consensus, inconsistencies across the dataset are possible. We only mapped interventions to the behavior change wheel (19), and not to the theoretical domain framework (23), because we believed that doing so retrospectively would involve too much ambiguity. We note that subjectivity of interpretation remains a real possibility, even as we encourage the application of behavioral theory to biosecurity intervention studies.

## 5 Conclusion

This review focused on reported efforts where the intention was to make a change in behavior relevant to disease prevention in agricultural animals. We first identified and classified interventions, finding a potential over-reliance on education as a driver of behavior. Subsequently, we mapped components of the interventions to the behavior change wheel and the COM-B framework, generating a modified framework that may prove useful in the context of biosecurity. We then followed our analysis with recommendations and considerations for those seeking to improve the adoption of biosecurity measures wherever livestock are concerned. We have provided a pathway for integrating behavior change wheel components into the design of biosecurity implementation studies. The mapping of interventions to COM-B and the behavior change wheel for this review was conducted retrospectively; however, it highlights the need for researchers to better describe their interventions even if they are not connected to specific theories of behavior change, and that following established guidance and checklists would be a major step towards improved intervention evaluation. By using intervention planning, program developers may be able to recognize earlier in the process where approaches are mismatched with objectives and adjust them accordingly to improve success. It is crucial to design studies rigorously when testing interventions. If only knowledge and self-reported (or audited) behaviors are measured, other potential drivers of behavior, such as attitudes, values, and resources cannot be identified as contributing influences (even if discussed later!). Research grounded in social ecological systems and focused on changing behavior, i.e., achieving the adoption of specific biosecurity measures (rather than changes in knowledge, attitudes, or scores), is needed to advance the field. Completed studies will have the greatest value if they follow standards for reporting on interventions, providing actionable insights into what worked and what did not, and why, to inform future efforts.

## Footnotes

1
https://osf.io/rxstj

2
https://food.ec.europa.eu/animals/animal-health/animal-health-law_en#information-material

3
https://www.poultryimprovement.org/

4
https://www.aphis.usda.gov/livestock-poultry-disease/swine/us-ship
