# Supplementary material for: How can we change behavior to bolster biosecurity? A scoping review and recommendations
Source: Front Vet Sci. 2026 Jul 22;13:1851630. doi: 10.3389/fvets.2026.1851630 (PMC13437398; doi:10.3389/fvets.2026.1851630)
Supplement: Supplementary file 1 [file Table_1.docx]

**Level 1 Screening: Title and Abstract Review**

Based on the title and abstract, use this set of questions to determine whether the record **describes biosecurity or disease prevention in relation to infectious disease of agricultural (or farmed or cultured) animals or livestock**? Refer to the definitions below for guidance on how we are interpreting these terms. If the answer to a question is unknown, select “maybe.” If the abstract is not attached to the record and the title of the record indicates the contents *may* meet the screening criteria, the reviewer should select “Maybe.” If the abstract is not attached to the record and the title of the record clearly does not meet the screening criteria, the reviewer should select “No.”

| Q1.  Is the record in English? | Yes, continue to Q2. | Unable to determine – select Maybe. Go to Q2. | No. Will exclude. Stop here. |
| --- | --- | --- | --- |
| Q2. Is this record a review or gap analysis (and therefore is not a primary source of research)? | No, continue to Q3. | Unable to determine – select Maybe. Go to Q3. | Yes  this record is clearly a review or gap analysis and not a primary source of research. Exclude. Stop here. |
| Q3.  Is this record about biosecurity or *infectious* disease prevention? (May use related terms.) | Yes, continue to Q4. | Unable to determine – select Maybe. Go to Q4. | No. Will exclude. Stop here. |
| Q4.  Is this record about a study that pertains to protecting the health of an agricultural or farmed (non-wildlife) or cultured (i.e., aquaculture) species? | Yes, this record will progress to level 2. | Unable to determine – select Maybe. | No. Exclude. Stop here. |
|  | Progress to level 2 | Progress to level 2 | Answers in this column will EXCLUDE the record from further consideration. |

Infectious disease: communicable or able to spread from one animal to another

Biosecurity: things people do to protect the health of animals, plants, and/or humans

Farmed or agricultural animals: species include terrestrial and aquatic, food and non-food animals (e.g. equids). For our purposes, terrestrial animals exclude wildlife. Agricultural animals include bees, but not other insects. (Domesticated bees are considered food animals.)
