# Supplementary material for: How can we change behavior to bolster biosecurity? A scoping review and recommendations
Source: Front Vet Sci. 2026 Jul 22;13:1851630. doi: 10.3389/fvets.2026.1851630 (PMC13437398; doi:10.3389/fvets.2026.1851630)
Supplement: Supplementary file 2 [file Table_2.docx]

**Level 2 Screening: Full Record Review**

Use the full record to answer the following questions. Only records that meet all eligibility criteria will proceed to level 3, data extraction.

| Q1. Is the full text available? | Yes | No  EXCLUDE |
| --- | --- | --- |
| Q2. Is the full text in English? | Yes | No  EXCLUDE |
| Q3. Is this a primary source*?  **A primary source will include data collected or collated during an investigation of an intervention. The investigation may be observational (for instance a case study or report) or experimental (in either a real or simulated setting).* | Yes | No, it is some sort of review or perspective or commentary  EXCLUDE |
| Q4. Does this work provide useful information**?  ***Useful information for the purpose of this review relates to an investigation whose objective was to assess the effectiveness of an intervention designed to change behavio!r at the production or management level to protect animal health. We are interested specifically in prevention of infectious (rather than production or metabolic) diseases in livestock, agricultural, or food animals, be they terrestrial or aquatic.* | Yes  INCLUDE  Go to Level 3 | No  EXCLUDE (and provide a reason)  The research is excluded because   (a) focus is biosafety (not at farm-level), bioterrorism, or biological weapons (b) focus is wildlife or insects (other than bees) (c) focus is upstream or downstream of production unit, i.e., feed safety or food safety (d) focus is human directed, either consumer-oriented or public health (e) focus is prevalence of a pathogen in relation to biosecurity (but not changing biosecurity to look for effect on prevalence of a pathogen) (f) focus is biosecurity measurement or assessment OR characterizing/understand-ing perceptions or attitudes or beliefs or other socio-psychological factors (but not of an intervention designed to change or influence factors or behavio!rs) (g) focus is programs or initiatives or tools but not their adoption or uptake |
