# Supplementary material for: How can we change behavior to bolster biosecurity? A scoping review and recommendations
Source: Front Vet Sci. 2026 Jul 22;13:1851630. doi: 10.3389/fvets.2026.1851630 (PMC13437398; doi:10.3389/fvets.2026.1851630)
Supplement: Supplementary file 3 [file Table_3.docx]

Supplementary Table 3

Study context and target audience of studies designed to change behavior at the production or management level to protect animal health.

| Reference | Location | Study Year | Livestock Type | Disease Focus | Target Stakeholders | No. of Participants |
| --- | --- | --- | --- | --- | --- | --- |
| (Agrawal et al., 2023) | Illinois, United States | 2021 | Swine | None | Pork producers | 13 |
| (Agrawal et al., 2023) | Illinois, United States | 2021 | Swine | None | Swine veterinarians | 7 |
| (Agrawal et al., 2023) | Illinois, United States | 2022 | Swine | None | Pork producers and veterinarians | 58 |
| (Agrawal et al., 2023) | Illinois, United States | 2022 | Swine | None | Pork producers and veterinarians | 0 – 26 per module |
| (Ahmed et al., 2021) | Dakahlia, Egypt | 2017 | Poultry | Avian influenza | Farm owners and workers | 244 |
| (Allen et al., 2019) | Washington, United States | Not specified | Beef cattle (cow-calf, i.e., suckler) | Bovine respiratory disease | Beef producers | 165 |
| (Amalraj et al., 2024) | Flanders and Wollonia, Belgium | 2022 - 2023 | Poultry | None | Broiler, layer, breeder, and turkey producers | 13 |
| (Bhandari et al., 2011) | Cambodia | Not specified | Poultry | None | Backyard poultry farm families | 100 |
| (Cardwell et al., 2016) | Southwest England and Wales, United Kingdom | 2008 - 2012 | Beef cattle (cow-calf, i.e., suckler) | BVD, IBR, L. hardjo, MAP, M. bovis | Beef producers | 46 of 57 (intervention); 47 of 59 (control) |
| (Chenais et al., 2023) | Gulu, Omoro, and Amuru, Uganda | 2019 – 2020 | Swine | African swine fever | Smallholder pig farmers and other stakeholders (“traders”) | 65 (59 and 54 at follow up visits) |

| Reference | Location | Study Year | Livestock Type | Disease Focus | Target Stakeholders | No. of Participants |
| --- | --- | --- | --- | --- | --- | --- |
| (Colvin et al., 2021) | Australia | Data from 2003, 2011, 2018 | Sheep | Parasites (internal and external) | Australia Wool Innovation wool producers | 1365 (2003); 575 (2011); 354 (2018) |
| (Conan, Luce Goutard, et al., 2013; Conan, Ponsich, et al., 2013) | Tram Kak and Samraong districts in Takeo province, Cambodia | 2009 – 2011 | Poultry (chickens, ducks) | None | Smallholder poultry farmers | Households in 9 intervention (372) and 9 control (377) villages |
| (Delpont et al., 2024) | Southwest France | 2022 | Poultry | None | Poultry farmers and poultry farm advisors; future farmers and future farm advisors of veterinarians | 123 total: 11 farmers, 19 farm advisors, 93 students |
| (Dione et al., 2020) | Lira and Masaka, Uganda | 2015 – 2017 | Swine | African swine fever | Smallholder pig farmers | 480 households initially assigned to each treatment |
| (Gillespie et al., 2021) | UK and worldwide (respondents from Canada, Europe, New Zealand, United Kingdom, and United States) | 2019 – 2020 | Dairy cattle | Bovine digital dermatitis | Dairy producers, hoof trimmers, and veterinarians | 143 (survey 1); 80 (survey 2) |

| Reference | Location | Study Year | Livestock Type | Disease Focus | Target Stakeholders | No. of Participants |
| --- | --- | --- | --- | --- | --- | --- |
| (Gosling et al., 2014) | England and Wales, United Kingdom | 2010 – 2011 | Poultry (egg-layers) | Salmonella | Commercial laying hen farmers or farm managers | 60 (initial); 59 (follow up) |
| (Grant et al., 2018) | England, United Kingdom | 2013 – 2014 | Sheep | Footrot | Sheep farmers with >300 sheep, >5% lameness, <3% contagious digital dermatitis | 29 of 32 |
| (Grant et al., 2018) | North, Midlands, and South of England, United Kingdom | 2013 – 2014 | Sheep | Footrot | Sheep farmers (that participated in 2013 survey) | 779 of 1081 |
| (Grant et al., 2018) | Southwest England, Wales, and English Marches, United Kingdom | 2013 – 2014 | Sheep | Footrot | Sheep farmer members of National Sheep Association | 51 of 78 (recruited out of 400) |
| (Komaladara et al., 2018) | Bali, Indonesia | 2015 | Poultry (broilers) | None | Companies that work with smallholder poultry farmers and participated in “Healthy Farm” trial | 6 |

| Reference | Location | Study Year | Livestock Type | Disease Focus | Target Stakeholders | No. of Participants |
| --- | --- | --- | --- | --- | --- | --- |
| (Liu et al., 2022) | United States | Not specified | Swine | None | Workers on Amazon Mechanical Turk role playing farm workers | 1592; 1305 English speakers, 287 Spanish speakers |
| (MacPhillamy et al., 2022) | Battambang, Siem Reap, Tbong Khmum, Kampong Cham, and Takeo, Cambodia | 2015 –2018 | Cattle or buffalo | Foot and mouth disease, hemorrhagic septicemia | Smallholder farmers with cattle (may also have pigs or poultry) | 240 (2015), 153 (2018) from 16 villages** |
| (MacPhillamy et al., 2022) | Xayabouli, Xieng Khoung, and Luang Prabang, Laos | 2015 –2018 | Cattle or buffalo | Foot and mouth disease, hemorrhagic septicemia | Smallholder farmers with cattle | 177 (2015), 133 (2018) from 9 villages** |
| (Marier et al., 2016) | Gloucester, Midlands, Suffolk, and Yorkshire (controls in Devon, Wiltshire, and Shropshire), England | 2008 – 2010 | Swine | Salmonella | Pig farmers | Four model farms, 8 or 9 surrounding farms for each model farm plus 9 control farms (i.e., 4 intervention farms, 33 contacts, and 9 "controls") |
| (Merrill et al., 2019) | Online, United States | Not specified | Swine | None | University student >18 YO | 118 |

| Reference | Location | Study Year | Livestock Type | Disease Focus | Target Stakeholders | No. of Participants |
| --- | --- | --- | --- | --- | --- | --- |
| (Merrill et al., 2019) | Online, United States | Not specified | Swine | None | University students and adults on Amazon Mechanical Turk (AMT) | 76, 102 |
| (Merrill et al., 2021) | Online, United States | Not specified | Swine | None | Adults on Amazon Mechanical Turk (AMT) | 140 (35 in each of 4 blocks) |
| (Nampanya et al., 2012) | Kampong Cham, Takeo, and Kandal, Cambodia | 2008 – 2010 | Cattle (and buffalo) | Foot-and-mouth disease, hemorrhagic septicemia, blackleg, internal and external parasites | Smallholder farmers with cattle | Six villages assigned to treatment or control; 150 respondents (25 per village) in 2008; 120 respondents (20 per village) in 2010 |
| (Raasch et al., 2020) same as (Collineau et al., 2017)) | Belgium, France, Germany, and Sweden | 2014 – 2015 | Swine (farrow to finish) | None (antimicrobial use) | Pig farmers with at least 70 sows and 500 market pigs per year | 68 farmers: 15 (Belgium), 19 (France), 25 (Germany), and 9 (Sweden) |

| Reference | Location | Study Year | Livestock Type | Disease Focus | Target Stakeholders | No. of Participants |
| --- | --- | --- | --- | --- | --- | --- |
| (Racicot et al., 2022) | Quebec, Canada | 2019 | Poultry (egg-layer and broiler) | None | Employees and visitors | 127 visits by 4 employees (layer), 28 visits by 3 employees (broiler) |
| (Racicot et al., 2022) | Quebec, Canada | 2021 – 2022 | Poultry (turkey) | None | Employees and visitors | 105 visits by 7 employees and 4 visitors |
| (Racicot et al., 2012) | Quebec, Canada | 2008 – 2010 | Poultry (broilers, layers, breeders) | None | Employees and visitors | 24 farms, 259 individuals (136 employees, 123 visitors) |
| (Rimi et al., 2016) | Chittagong and Rajshahi, Bangladesh | 2009 | Poultry | HPAI | 138 (Chittagong) and 114 (Rajshahi) households, most with backyard poultry | 42 before, 36 after |
| (Schreuder et al., 2023) | Netherlands, Greece, and Cyprus | 2019 – 2021 | Poultry (broilers) | None | Poultry farmers | 35 broiler houses on 30 farms |
| (Scollo et al., 2022) | Northern Italy | Not specified | Swine (growers/fattening) | None | Pig farmers within same contract structure | 15 sites |
| (Scollo et al., 2023) | Piedmont, Lombardy, and Emilia Romagna (northern Italy) | Not specified | Swine (farrow-to-wean/breeding-nursery, finishers/fattening) | None | Farmers and employees from farms with same contract agreements | 29 farm staff from 20 farms |
| Reference | Location | Study Year | Livestock Type | Disease Focus | Target Stakeholders | No. of Participants |
| (Smith et al., 2014) | California, United States | Not specified | 4-H livestock projects | None | 4-H youth | Survey data from 54 of 120 (level 1); 50 of 104 (level 2); 25 of 86 (level 3); 23 of 63 (level 4)*** |
| (Smith et al., 2014) | California, United States | Not specified | 4-H livestock projects | None | Adults | 63 of 140 adult community members |
| (Tilli et al., 2024) | Veneto, Lombardia, Emilia Romagna, Piemonte, and Puglia, Italy | 2021 | Poultry (chickens, turkeys, ducks), broilers, egg-layers, or breeders | None | Farmers and advisors (vets, academics, producer organisations) | 30 farmers, 37 advisors |
| (Tilli et al., 2024) | Veneto, Lombardia, Emilia Romagna, Piemonte, and Puglia, Italy | 2022 – 2023 | Poultry (chickens, turkeys, ducks), broilers, egg-layers, or breeders | None | Poultry farmers or farm managers or other | 26 of 29 completed validation |
| (Trinity et al., 2020) | Online, United States | Not specified | Swine | None | Adults on Amazon Mechanical Turk (AMT) | 108 |

References

Agrawal, I., Bromfield, C., & Varga, C. (2023). Assessing and improving on-farm biosecurity knowledge and practices among swine producers and veterinarians through online surveys and an educational website in Illinois, United States. *Frontiers in Veterinary Science*, *10*, Article 1167056. <https://doi.org/10.3389/fvets.2023.1167056>

Ahmed, H. A., Mohamed, M. E. M., Erfan, A. M., Abdelkarim, L., & Awadallah, M. A. I. (2021). Investigating the biosecurity measures' applications in poultry farms and its relationship with the occurence of avian influenza. *Slovenian Veterinary Research*, *58*, 315-321. <https://doi.org/10.26873/SVR-1451-2021>

Allen, A. J., Neibergs, J. S., Llewellyn, D. A., Smith, S. M., Kerr, S. R., Moore, D. A., & Hudson, T. D. (2019). Developing herd health education for and assessing risky practices of cow-calf producers. *Journal of Extension*, *57*(5), Article 16. <https://doi.org/10.34068/joe.57.05.16>

Amalraj, A., Van Meirhaeghe, H., Chantziaras, I., & Dewulf, J. (2024). Assessing the impact of on-farm biosecurity coaching on farmer perception and farm biosecurity status in Belgian poultry production. *Animals*, *14*(17), Article 2498. <https://doi.org/10.3390/ani14172498>

Bhandari, D. P., Wollen, T. S., & Lohani, M. N. (2011). Preventing highly pathogenic avian influenza (HPAI) at the rural community level: a case study from Cambodia. *Tropical Animal Health and Production*, *43*(6), 1071-1073. <https://doi.org/10.1007/s11250-011-9828-y>

Cardwell, J. M., Van Winden, S., Beauvais, W., Mastin, A., De Glanville, W. A., Hardstaff, J., Booth, R. E., Fishwick, J., & Pfeiffer, D. U. (2016). Assessing the impact of tailored biosecurity advice on farmer behaviour and pathogen presence in beef herds in England and Wales. *Preventive Veterinary Medicine*, *135*, 9-16. <https://doi.org/10.1016/j.prevetmed.2016.10.018>

Chenais, E., Fischer, K., Aliro, T., Ståhl, K., & Lewerin, S. S. (2023). Co-created community contracts support biosecurity changes in a region where African swine fever is endemic-Part II: Implementation of biosecurity measures. *Preventive Veterinary Medicine*, *214*, Article 105902. <https://doi.org/10.1016/j.prevetmed.2023.105902>

Collineau, L., Rojo-Gimeno, C., Leger, A., Backhans, A., Loesken, S., Nielsen, E. O., Postma, M., Emanuelson, U., Beilage, E. G., Sjolund, M., Wauters, E., Stark, K. D. C., Dewulf, J., Belloc, C., & Krebs, S. (2017). Herd-specific interventions to reduce antimicrobial usage in pig production without jeopardising technical and economic performance. *Preventive Veterinary Medicine*, *144*, 167-178. <https://doi.org/10.1016/j.prevetmed.2017.05.023>

Colvin, A. F., Reeve, I., Thompson, L. J., Kahn, L. P., & Walkden-Brown, S. W. (2021). Australian surveys on parasite control in sheep between 2003 and 2019 reveal marked regional variation and increasing utilisation of online resources and on-farm biosecurity practices. *Veterinary Parasitology: Regional Studies and Reports*, *25*, Article 100614. <https://doi.org/10.1016/j.vprsr.2021.100614>

Conan, A., Luce Goutard, F., Holl, D., Ra, S., Ponsich, A., Tarantola, A., Sorn, S., & Vong, S. (2013). Cluster randomised trial of the impact of biosecurity measures on poultry health in backyard flocks. *Veterinary Journal*, *198*(3), 649-655. <https://doi.org/10.1016/j.tvjl.2013.09.010>

Conan, A., Ponsich, A., Luce Goutard, F., Khiev, R., Tarantola, A., Sorn, S., & Vong, S. (2013). A community-based education trial to improve backyard poultry biosecurity in rural Cambodia. *Acta Tropica*, *125*(3), 294-302. <https://doi.org/10.1016/j.actatropica.2012.12.006>

Delpont, M., Chavoix, C., Fourtune, L., Durivage, A., Guérin, J. L., Vaillancourt, J. P., & Paul, M. C. (2024). Making the invisible visible: Effectiveness of a training program to increase awareness and biosecurity compliance in poultry barn anterooms, using glowing substances. *Preventive Veterinary Medicine*, *233*, Article 106349. <https://doi.org/10.1016/j.prevetmed.2024.106349>

Dione, M. M., Dohoo, I., Ndiwa, N., Poole, J., Ouma, E., Amia, W. C., & Wieland, B. (2020). Impact of participatory training of smallholder pig farmers on knowledge, attitudes and practices regarding biosecurity for the control of African swine fever in Uganda. *Transboundary and Emerging Diseases*, *67*(6), 2482-2493. <https://doi.org/10.1111/tbed.13587>

Gillespie, A. V., Carter, S. D., Blowey, R. W., Staton, G. J., Walsh, T. R., & Evans, N. J. (2021). Measuring the impact of bovine digital dermatitis research on knowledge and practice of biosecurity during cattle foot-trimming. *Journal of Dairy Research*, *88*(1), 60-63, Article Pii s0022029921000170. <https://doi.org/10.1017/S0022029921000170>

Gosling, R. J., Martelli, F., Wintrip, A., Sayers, A. R., Wheeler, K., & Davies, R. H. (2014). Assessment of producers' response to Salmonella biosecurity issues and uptake of advice on laying hen farms in England and Wales. *British Poultry Science*, *55*(5), 559-568. <https://doi.org/10.1080/00071668.2014.949620>

Grant, C., Kaler, J., Ferguson, E., O'Kane, H., & Green, L. E. (2018). A comparison of the efficacy of three intervention trial types: postal, group, and one-to-one facilitation, prior management and the impact of message framing and repeat messages on the flock prevalence of lameness in sheep. *Preventive Veterinary Medicine*, *149*, 82-91. <https://doi.org/10.1016/j.prevetmed.2017.11.013>

Komaladara, A., Patrick, I., & Hoang, N. (2018). Contract bonus systems to encourage biosecurity adoption on small-scale broiler farms in Indonesia. *Animal Production Science*, *58*(3), 595-600. <https://doi.org/10.1071/AN15845>

Liu, T.-L., Merrill, S. C., O'Keefe, A., Clark, E. M., Langle-Chimal, O. D., Trinity, L., Shrum, T. R., Koliba, C., Zia, A., Sellnow, T. L., Sellnow, D. D., & Smith, J. M. (2022). Effects of message delivery on cross-cultural biosecurity compliance: Insights from experimental simulations. *Frontiers in Veterinary Science*, *9*, Article 984945. <https://doi.org/10.3389/fvets.2022.984945>

MacPhillamy, I., Olmo, L., Young, J., Nampanya, S., Suon, S., Khounsy, S., Windsor, P., Toribio, J.-A., & Bush, R. (2022). Changes in farmer animal health and biosecurity knowledge, attitudes and practices: Insights from Cambodia and Laos. *Transboundary and Emerging Diseases*, *69*(4), e517-e531. <https://doi.org/10.1111/tbed.14328>

Marier, E., Piers Smith, R., Ellis-Iversen, J., Watson, E., Armstrong, D., Hogeveen, H., & Cook, A. J. (2016). Changes in perceptions and motivators that influence the implementation of on-farm *Salmonella* control measures by pig farmers in England. *Preventive Veterinary Medicine*, *133*, 22-30. <https://doi.org/10.1016/j.prevetmed.2016.09.009>

Merrill, S. C., Moegenburg, S., Koliba, C. J., Zia, A., Trinity, L., Clark, E., Bucini, G., Wiltshire, S., Sellnow, T., Sellnow, D., & Smith, J. M. (2019). Willingness to comply with biosecurity in livestock facilities: Evidence from experimental simulations. *Frontiers in Veterinary Science*, *6*, 156. <https://doi.org/10.3389/fvets.2019.00156>

Merrill, S. C., Trinity, L., Clark, E. M., Shrum, T. R., Koliba, C. J., Zia, A., Bucini, G., Sellnow, T. L., Sellnow, D. D., & Smith, J. M. (2021). Message delivery strategy influences willingness to comply with biosecurity. *Frontiers in Veterinary Science*, *8*, 667265. <https://doi.org/10.3389/fvets.2021.667265>

Nampanya, S., Suon, S., Rast, L., & Windsor, P. A. (2012). Improvement in smallholder farmer knowledge of cattle production, health and biosecurity in Southern Cambodia between 2008 and 2010. *Transboundary and Emerging Diseases*, *59*(2), 117-127. <https://doi.org/10.1111/j.1865-1682.2011.01247.x>

Raasch, S., Collineau, L., Postma, M., Backhans, A., Sjolund, M., Belloc, C., Emanuelson, U., Beilage, E. G., Stark, K., Dewulf, J., & on the behalf of the, M. C. (2020). Effectiveness of alternative measures to reduce antimicrobial usage in pig production in four European countries. *Porcine Health Management*, *6*, 6. <https://doi.org/10.1186/s40813-020-0145-6>

Racicot, M., Cardinal, A. M., Tremblay, D., & Vaillancourt, J.-P. (2022). Technologies monitoring and improving biosecurity compliance in barn anterooms. *Frontiers in Veterinary Science*, *9*, Article 1005144. <https://doi.org/10.3389/fvets.2022.1005144>

Racicot, M., Venne, D., Durivage, A., & Vaillancourt, J.-P. (2012). Evaluation of strategies to enhance biosecurity compliance on poultry farms in Quebec: effect of audits and cameras. *Preventive Veterinary Medicine*, *103*(2-3), 208-218. <https://doi.org/10.1016/j.prevetmed.2011.08.004>

Rimi, N. A., Sultana, R., Ishtiak-Ahmed, K., Rahman, M. Z., Hasin, M., Islam, M. S., Azziz-Baumgartner, E., Nahar, N., Gurley, E. S., & Luby, S. P. (2016). Understanding the failure of a behavior change intervention to reduce risk behaviors for avian influenza transmission among backyard poultry raisers in rural Bangladesh: a focused ethnography. *BMC Public Health*, *16*(1), 858. <https://doi.org/10.1186/s12889-016-3543-6>

Schreuder, J., Simitopoulou, M., Angastiniotis, K., Ferrari, P., Wolthuis-Fillerup, M., Kefalas, G., & Papasolomontos, S. (2023). Development and implementation of a risk assessment tool for broiler farm biosecurity and a health intervention plan in the Netherlands, Greece, and Cyprus [Article]. *Poultry Science*, *102*(2), Article 102394. <https://doi.org/10.1016/j.psj.2022.102394>

Scollo, A., Levallois, P., Fourichon, C., Motta, A., Mannelli, A., Lombardo, F., & Ferrari, P. (2022). Monitoring means and results of biosecurity in pig fattening farms: Systematic assessment of measures in place and exploration of biomarkers of interest. *Animals*, *12*(19), Article 2655. <https://doi.org/10.3390/ani12192655>

Scollo, A., Perrucci, A., Stella, M. C., Ferrari, P., Robino, P., & Nebbia, P. (2023). Biosecurity and hygiene procedures in pig farms: Effects of a tailor-made approach as monitored by environmental samples. *Animals*, *13*(7), Article 1262. <https://doi.org/10.3390/ani13071262>

Smith, M. H., Meehan, C. L., & Borba, J. A. (2014). Bio-Security proficiencies project for beginning producers in 4-H. *Journal of Extension*, *52*(6), Article 2. <https://doi.org/10.34068/joe.52.06.02>

Tilli, G., Laconi, A., Galuppo, F., Grilli, G., Zbikowski, A., Amalraj, A., & Piccirillo, A. (2024). Supporting measures to improve biosecurity within Italian poultry production. *Animals*, *14*(12), Article 1734. <https://doi.org/10.3390/ani14121734>

Trinity, L., Merrill, S. C., Clark, E. M., Koliba, C. J., Zia, A., Bucini, G., & Smith, J. M. (2020). Effects of social cues on biosecurity compliance in livestock facilities: Evidence from experimental simulations. *Frontiers in Veterinary Science*, *7*, Article 130. <https://doi.org/10.3389/fvets.2020.00130>
