# Supplementary material for: How can we change behavior to bolster biosecurity? A scoping review and recommendations
Source: Front Vet Sci. 2026 Jul 22;13:1851630. doi: 10.3389/fvets.2026.1851630 (PMC13437398; doi:10.3389/fvets.2026.1851630)
Supplement: Supplementary file 4 [file Table_4.docx]

Supplementary Table 4

Additional descriptions of studies designed to change behavior at the production or management level to protect animal health.

| Reference | Length of Study | Intervention Activity | Evaluation Comparator | Outcome Indicator | Theoretical Framework |
| --- | --- | --- | --- | --- | --- |
| (Agrawal et al., 2023) | 4 weeks | Survey | None | Knowledge, perceptions, practice (K, B, P) | Health belief model indicated but no details given |
| (Agrawal et al., 2023) | 8 weeks | Survey | None | Knowledge, perceptions, practice (K, B, P) | ibid |
| (Agrawal et al., 2023) | July – November 2022 | Infographics on website | None | Downloads (P) | ibid |
| (Agrawal et al., 2023) | July – November 2022 | Educational website | None | Web traffic, voluntary post-module quiz scores (P, K) | ibid |
| (Ahmed et al., 2021) | Second visit three months after first | Advice at end of first visit | Within farm pre-post (second vs first visit) | Biosecurity practices and occurrence of avian influenza (P, H) | None specified |
| (Allen et al., 2019) | One-day workshop | In-person workshop and voluntary self-assessment | Within subject pre vs post (test responses) | Short-term knowledge gain (K) | None specified |
| (Amalraj et al., 2024) | Three in-person visits within a year | Personalized advice at second visit and coaching next six months | Third vs first visit | ADKAR and Biocheck scores (K, B) | ADKAR: awareness, desire, knowledge, ability, reinforcement |

| Reference | Length of Study | Intervention Activity | Evaluation Comparator | Outcome Indicator | Theoretical Framework |
| --- | --- | --- | --- | --- | --- |
| (Bhandari et al., 2011) | 2 years | Village animal health workers were trained and worked with model farms which then served as demonstration sites | Participatory self-review | Biosecurity and disease incidence (P, H) | Participatory action framework (Heifer cornerstones) |
| (Cardwell et al., 2016) | Four years | Annual risk assessment and detailed, tailored biosecurity advice | Annual risk assessment and generic advice | Risk scores and seroprevalence (O, H) | None specified but approach similar to competing values framework |
| (Chenais et al., 2023) | Follow up meetings 4 and 6 months after first | Farmer and trader subgroups in each community identified biosecurity measures to implement | Baseline implementation recorded at first meeting | Implementation of biosecurity measures between first and other meetings (P) | Participatory co-creation approach |
| (Colvin et al., 2021) | Three survey rounds (2004, 2012, and 2019) | IPM-s project, Paraboss website, and industry surveys | 2003 data (collected in 2004) | Use of Paraboss website (P) | None specified |
| (Conan, Luce Goutard, et al., 2013; Conan, Ponsich, et al., 2013) | 15 months (4 months baseline, train-the-trainer, and trainings; 11 months follow up) | Educational package distributed by trained community teams (intervention villages) OR village teams raised awareness of hand and food hygiene (without providing training) | Cluster randomized trial with two arms; Comparisons made within intervention villages or between intervention and control (depending on the type of data) | Information transmission, change in knowledge, and change in practice (Conan Acta); Mortality rates and HPAI and ND serological results (Conan TVJL) (K, P, H) | None specified |
| Reference | Length of Study | Intervention Activity | Evaluation Comparator | Outcome Indicator | Theoretical Framework |
| (Delpont et al., 2024) | Twelve two-three hour assessment and training sessions from July through October | Socio-demographic, attitude, and risk perception questionnaires, training exercises, identification of errors in videos, 2nd attitude survey | Within subject pre-post | Attitude change (K, A, B, S) | Ajzen’s theory of planned behavior with focus on targeting attitudes |
| (Dione et al., 2020) | Initial survey and four-hour training session April 2015; follow up survey in May 2016 and September 2017 | Four-hour initial training; refresher training three months later in treatment villages | Baseline KAP survey in April 2015 compared with survey in May 2016; treatment group surveyed again in September 2017 | Within and between treatment change in KAP at 12 months; within treatment change also measured at 28 months (K, B, P) | Item response theory model |
| (Gillespie et al., 2021) | Surveys conducted in 2019 and 2020 | Foot-trimming hygiene protocol disseminated through multiple channels and online | Surveys before and after promotion of hygiene protocol (not necessarily same respondents) | Awareness of protocol and hygiene measures practiced (K, P) | Loosely based on theory of reasoned action |
| (Gosling et al., 2014) | August 2010 – September 2011; Two to nine months between visits | Audit, selected recommendation cards, follow up visit 2 - 9 mo later | Pre and post assessments | Biosecurity measures in place (P) | None specified |

| Reference | Length of Study | Intervention Activity | Evaluation Comparator | Outcome Indicator | Theoretical Framework |
| --- | --- | --- | --- | --- | --- |
| (Grant et al., 2018) | Not specified | Knowledge-transfer tactic: one-on-one consultation, engagement in adopting program, and follow up written advice | Within flock change | Change in prevalence of lameness and participants' behavior (measured in follow up postal survey) (P, H) | Message framing, tailoring of message |
| (Grant et al., 2018) | Not specified | Knowledge-transfer tactic: postal 7 arm trial; control received intervention message at end of trial, other "arms" received loss vs gain messages on different intervals (once, three times, or seasonally) | Within flock and between flock | Change in prevalence of lameness and participants' behavior (measured in follow up postal survey) (P, H) | Message framing |
| (Grant et al., 2018) | Not specified | Knowledge-transfer tactic: 2 meetings with different framing in each of 3 regions; loss vs gain framing | Within flock change | Change in prevalence of lameness and participants' behavior (measured in follow up postal survey) (P, H) | Message framing |
| (Komaladara et al., 2018) | Not applicable | Analysis of contracts | Between contract gross margin analysis | Change in gross margin, and return on investment in biosecurity adoption to identify type of contract that best supports biosecurity implementation (O) | Economic incentive as motivation |
| Reference | Length of Study | Intervention Activity | Evaluation Comparator | Outcome Indicator | Theoretical Framework |
| (Liu et al., 2022) | About 30 minutes to complete training and 24 rounds of experiment | 24 rounds of experimental simulation game presenting infection risk information in different formats and either English or Spanish | Among treatments and by language: message delivery methods, infection risk levels, and certain vs uncertain messaging conditions | Compliance in game (i.e., use shower-in shower-out as risk increases) (P) | Hofstede's cultural dimensions theory (particularly uncertainty avoidance index), prospect theory (loss aversion), cross-cultural risk perception theories |
| (MacPhillamy et al., 2022) | Biosecurity KAP surveys July 2015 and May 2018; Formal training during first 18 months of project followed by on-the-job training | Randomly assigned villages to intervention (vacc & training) or control (vacc only); formal and on-the-job training | Pre and post surveys | Knowledge change and influence of knowledge on attitudes and practice; Intercluster coefficient (K, A, P) | Knowledge-attitude-practice (KAP) model; Consideration of resource scarcity and poverty’s cognitive burden in decision-making |

| Reference | Length of Study | Intervention Activity | Evaluation Comparator | Outcome Indicator | Theoretical Framework |
| --- | --- | --- | --- | --- | --- |
| (MacPhillamy et al., 2022) | Biosecurity KAP surveys November 2015 and May 2018; Formal training during first 18 months of project followed by on-the-job training | Randomly assigned villages to intervention (vacc & training) or control (vacc only) or control (biosecurity training only); formal and on-the-job training | Pre and post surveys | Knowledge change and influence of knowledge on attitudes and practice; Intercluster coefficient (K, A, P) | Knowledge-attitude-practice (KAP) model; Consideration of socio-economic context as in 19a |
| (Marier et al., 2016) | Initial visits August 2008 – May 2009; final visits June – October 2010 | Each model farm implemented a different interven-tion (BioMos in sow rations, form of grower feed, Salmonella vaccin-ation of sows plus acidified feed of young pigs, or Salmonella vaccin-ation of piglets); contact farms were not directly involved; controls were not connected with pilot or contact farms | Within farm pre-post; between farm controls vs contacts | Perceptions of effectiveness, attitudes, perceptions, barriers, motivators; ZNCP scores, if available (A, B, H) | Pathway to disease control model (an extension of the theory of reasoned behavior) |

| Reference | Length of Study | Intervention Activity | Evaluation Comparator | Outcome Indicator | Theoretical Framework |
| --- | --- | --- | --- | --- | --- |
| (Merrill et al., 2019) | Not specified | 24 rounds (each representing one day); information provided in simulated pig barn to accept risk of disease by exiting without appropriate biosecurity vs opportunity costs associated with the decision to use biosecurity (showering in/out). risk level (3), risk uncertainty, information delivery method (2) | Complete block design; mixed effects logistic regression | Odds of compliance in game (use of shower-in shower-out as risk increases) (P) | Behavioral economics; accounted for play order (learning), effect of infection (psychological distancing) |
| (Merrill et al., 2019) | Not specified | 24 rounds (each representing one day); information provided in simulated pig barn to accept risk of disease by exiting without appropriate biosecurity vs opportunity costs associated with the decision to use biosecurity (showering in/out). risk level (4), uncertainty of risk, information delivery method (3) | Complete block design; mixed effects logistic regression | Odds of compliance in game (use of shower-in shower-out as risk increases) (P) | Behavioral economics; accounted for play order (learning), effect of infection (psychological distancing), and audience (payment incentive effect between students and AMT) |

| Reference | Length of Study | Intervention Activity | Evaluation Comparator | Outcome Indicator | Theoretical Framework |
| --- | --- | --- | --- | --- | --- |
| (Merrill et al., 2021) | Not specified | 24 rounds (each representing a day) of experimental simulation representing 75% of 32 total treatments; risk communication message format (linguistic, numeric, linguistic or numeric threat gauge), infection risk (1, 5, 15, 25%), infection uncertainty | Incomplete block design; effect of treatment levels on decision to shower in -shower out analyzed by mixed effects logistic regression model | Decision to choose shower vs emergency exit (P) | Socio-psychological determinants of motivation, nudges |
| (Nampanya et al., 2012) | Ongoing activities over 2.5 years | High intervention: Vaccination/ participatory applied field research, on-the-job training, and formal training in three villages; Low intervention: Vaccination/ participatory applied field research only in three villages**** | High vs low intervention villages; pre and post surveys | Change in knowledge and attitudes (K, A) | Knowledge-based intervention theory; Agricultural extension approaches (participatory research, on-the-job training, formal training); Integrated development approach (combining productivity, health, and marketing interventions) |
| Reference | Length of Study | Intervention Activity | Evaluation Comparator | Outcome Indicator | Theoretical Framework |
| (Raasch et al., 2020) same as (Collineau et al., 2017) | baseline December 2012 – December 2013; intervention February 2014 – August 2015 | Advisory team developed herd-specific plan in consultation with farmer, farmer committed to follow plan; herds visited 2 to 6 times over next year, farmers in two countries received compensation | One year retrospective vs one year prospective | Compliance score, change in treatment incidence, disease incidence, cost; Within farm (biosecurity practices) and between farms (animal performance) (P, H) | None specified |
| (Racicot et al., 2022) | 17 days (February – March, egg-layer); 13 days (May – June, broiler) | MediHandTrace® RFID chip system with alarm | Data from previous project (2012) | Compliance with boot and hand hygiene and attitudes about system (A, P) | None specified |
| (Racicot et al., 2022) | 1 month (December – January) | Maximus® pressure mat system with or without alarm (2 weeks each) | Simultaneous video recording | Compliance with boot and hand hygiene (P) | None specified |
| (Racicot et al., 2012) | 2 weeks of monitoring, at beginning and six months later | Assigned to control (sign and hidden camera only), audit (sign, hidden camera and three audits over six months), or camera (sign and hidden camera, notified of constant monitoring) | Control vs treatment and short-term vs medium-term | Proportion of visits where biosecurity measure respected; compliance with stated biosecurity measures (P) | None specified |
| Reference | Length of Study | Intervention Activity | Evaluation Comparator | Outcome Indicator | Theoretical Framework |
| (Rimi et al., 2016) | Two months (one month before and after intervention) between June and August | Information campaign—village meetings and illustrated posters | Pre and post intervention observations and interviews | Changes in awareness and practices (K, P) | Ethnographic approach |
| (Schreuder et al., 2023) | 16 months (4 broiler production cycles) | Farm-specific health plan based on systematic biosecurity risk assessment implemented after second production cycle, evaluated after fourth cycle | Within farm pre-post (two cycles before and two cycles after implementation of plan) | Implementation of recommended measures (P) | None specified |
| (Scollo et al., 2022) | Not specified | Site-specific biosecurity plan based on systematic biosecurity risk assessment, audited 6 and 12 months after plan developed | Within site pre-post | Change in biosecurity score; change in biomarkers (P, H) | None specified |
| (Scollo et al., 2023) | One year; BEAT visit, 6-mo visit, and 12-mo visit | Systematic biosecurity risk assessment, training on hygiene, follow-up assessments and sampling | Within farm pre vs post | Biosecurity scores; ATP, ESBL E. coli, and LA-MRSA test results (P, H) | None specified |

| Reference | Length of Study | Intervention Activity | Evaluation Comparator | Outcome Indicator | Theoretical Framework |
| --- | --- | --- | --- | --- | --- |
| (Smith et al., 2014) | Not specified | Completion of the Biosecurity Proficiencies Project for Beginning Producers | Retrospective post as pre | Change in knowledge and skills (K, S) | None specified; applied good pedagogy |
| (Smith et al., 2014) | Not specified | Attendance at public presentation by youth in biosecurity proficiencies project | Retrospective post as pre | Change in knowledge and skills (K, S) | None specified |
| (Tilli et al., 2024) | Not specified | Survey | None (observational) | Potential supportive measures to pilot (O) | None specified |
| (Tilli et al., 2024) | Not specified | Biocheck and coaching | Within farm pre-post | Implementation of action plan measures (P) | ADKAR (awareness, desire, know-ledge, ability, reinforcement) |
| (Trinity et al., 2020) | Not specified | 18 rounds; virtual production facility with variables such as risk of infection associated with noncompliance, delivery method of infection risk message, and behavior of co-worker | Risk level (low, med, high), message type (numeric, threat gauge) and social cues (compliance, non-compliance of simulated co-worker) | Compliance with biosecurity in simulation (i.e., use of shower-in shower-out as risk increased) (P) | Socio-psychological behavioral determinants: social cues, psychological distance, affective reasoning |

A – attitude; B – belief, risk perception; H – health outcome; K – knowledge; O – other; P – behavior, practice; S – skill

References

Agrawal, I., Bromfield, C., & Varga, C. (2023). Assessing and improving on-farm biosecurity knowledge and practices among swine producers and veterinarians through online surveys and an educational website in Illinois, United States. *Frontiers in Veterinary Science*, *10*, Article 1167056. <https://doi.org/10.3389/fvets.2023.1167056>

Ahmed, H. A., Mohamed, M. E. M., Erfan, A. M., Abdelkarim, L., & Awadallah, M. A. I. (2021). Investigating the biosecurity measures' applications in poultry farms and its relationship with the occurence of avian influenza. *Slovenian Veterinary Research*, *58*, 315-321. <https://doi.org/10.26873/SVR-1451-2021>

Allen, A. J., Neibergs, J. S., Llewellyn, D. A., Smith, S. M., Kerr, S. R., Moore, D. A., & Hudson, T. D. (2019). Developing herd health education for and assessing risky practices of cow-calf producers. *Journal of Extension*, *57*(5), Article 16. <https://doi.org/10.34068/joe.57.05.16>

Amalraj, A., Van Meirhaeghe, H., Chantziaras, I., & Dewulf, J. (2024). Assessing the impact of on-farm biosecurity coaching on farmer perception and farm biosecurity status in Belgian poultry production. *Animals*, *14*(17), Article 2498. <https://doi.org/10.3390/ani14172498>

Bhandari, D. P., Wollen, T. S., & Lohani, M. N. (2011). Preventing highly pathogenic avian influenza (HPAI) at the rural community level: a case study from Cambodia. *Tropical Animal Health and Production*, *43*(6), 1071-1073. <https://doi.org/10.1007/s11250-011-9828-y>

Cardwell, J. M., Van Winden, S., Beauvais, W., Mastin, A., De Glanville, W. A., Hardstaff, J., Booth, R. E., Fishwick, J., & Pfeiffer, D. U. (2016). Assessing the impact of tailored biosecurity advice on farmer behaviour and pathogen presence in beef herds in England and Wales. *Preventive Veterinary Medicine*, *135*, 9-16. <https://doi.org/10.1016/j.prevetmed.2016.10.018>

Chenais, E., Fischer, K., Aliro, T., Ståhl, K., & Lewerin, S. S. (2023). Co-created community contracts support biosecurity changes in a region where African swine fever is endemic-Part II: Implementation of biosecurity measures. *Preventive Veterinary Medicine*, *214*, Article 105902. <https://doi.org/10.1016/j.prevetmed.2023.105902>

Collineau, L., Rojo-Gimeno, C., Leger, A., Backhans, A., Loesken, S., Nielsen, E. O., Postma, M., Emanuelson, U., Beilage, E. G., Sjolund, M., Wauters, E., Stark, K. D. C., Dewulf, J., Belloc, C., & Krebs, S. (2017). Herd-specific interventions to reduce antimicrobial usage in pig production without jeopardising technical and economic performance. *Preventive Veterinary Medicine*, *144*, 167-178. <https://doi.org/10.1016/j.prevetmed.2017.05.023>

Colvin, A. F., Reeve, I., Thompson, L. J., Kahn, L. P., & Walkden-Brown, S. W. (2021). Australian surveys on parasite control in sheep between 2003 and 2019 reveal marked regional variation and increasing utilisation of online resources and on-farm biosecurity practices. *Veterinary Parasitology: Regional Studies and Reports*, *25*, Article 100614. <https://doi.org/10.1016/j.vprsr.2021.100614>

Conan, A., Luce Goutard, F., Holl, D., Ra, S., Ponsich, A., Tarantola, A., Sorn, S., & Vong, S. (2013). Cluster randomised trial of the impact of biosecurity measures on poultry health in backyard flocks. *Veterinary Journal*, *198*(3), 649-655. <https://doi.org/10.1016/j.tvjl.2013.09.010>

Conan, A., Ponsich, A., Luce Goutard, F., Khiev, R., Tarantola, A., Sorn, S., & Vong, S. (2013). A community-based education trial to improve backyard poultry biosecurity in rural Cambodia. *Acta Tropica*, *125*(3), 294-302. <https://doi.org/10.1016/j.actatropica.2012.12.006>

Delpont, M., Chavoix, C., Fourtune, L., Durivage, A., Guérin, J. L., Vaillancourt, J. P., & Paul, M. C. (2024). Making the invisible visible: Effectiveness of a training program to increase awareness and biosecurity compliance in poultry barn anterooms, using glowing substances. *Preventive Veterinary Medicine*, *233*, Article 106349. <https://doi.org/10.1016/j.prevetmed.2024.106349>

Dione, M. M., Dohoo, I., Ndiwa, N., Poole, J., Ouma, E., Amia, W. C., & Wieland, B. (2020). Impact of participatory training of smallholder pig farmers on knowledge, attitudes and practices regarding biosecurity for the control of African swine fever in Uganda. *Transboundary and Emerging Diseases*, *67*(6), 2482-2493. <https://doi.org/10.1111/tbed.13587>

Gillespie, A. V., Carter, S. D., Blowey, R. W., Staton, G. J., Walsh, T. R., & Evans, N. J. (2021). Measuring the impact of bovine digital dermatitis research on knowledge and practice of biosecurity during cattle foot-trimming. *Journal of Dairy Research*, *88*(1), 60-63, Article Pii s0022029921000170. <https://doi.org/10.1017/S0022029921000170>

Gosling, R. J., Martelli, F., Wintrip, A., Sayers, A. R., Wheeler, K., & Davies, R. H. (2014). Assessment of producers' response to Salmonella biosecurity issues and uptake of advice on laying hen farms in England and Wales. *British Poultry Science*, *55*(5), 559-568. <https://doi.org/10.1080/00071668.2014.949620>

Grant, C., Kaler, J., Ferguson, E., O'Kane, H., & Green, L. E. (2018). A comparison of the efficacy of three intervention trial types: postal, group, and one-to-one facilitation, prior management and the impact of message framing and repeat messages on the flock prevalence of lameness in sheep. *Preventive Veterinary Medicine*, *149*, 82-91. <https://doi.org/10.1016/j.prevetmed.2017.11.013>

Komaladara, A., Patrick, I., & Hoang, N. (2018). Contract bonus systems to encourage biosecurity adoption on small-scale broiler farms in Indonesia. *Animal Production Science*, *58*(3), 595-600. <https://doi.org/10.1071/AN15845>

Liu, T.-L., Merrill, S. C., O'Keefe, A., Clark, E. M., Langle-Chimal, O. D., Trinity, L., Shrum, T. R., Koliba, C., Zia, A., Sellnow, T. L., Sellnow, D. D., & Smith, J. M. (2022). Effects of message delivery on cross-cultural biosecurity compliance: Insights from experimental simulations. *Frontiers in Veterinary Science*, *9*, Article 984945. <https://doi.org/10.3389/fvets.2022.984945>

MacPhillamy, I., Olmo, L., Young, J., Nampanya, S., Suon, S., Khounsy, S., Windsor, P., Toribio, J.-A., & Bush, R. (2022). Changes in farmer animal health and biosecurity knowledge, attitudes and practices: Insights from Cambodia and Laos. *Transboundary and Emerging Diseases*, *69*(4), e517-e531. <https://doi.org/10.1111/tbed.14328>

Marier, E., Piers Smith, R., Ellis-Iversen, J., Watson, E., Armstrong, D., Hogeveen, H., & Cook, A. J. (2016). Changes in perceptions and motivators that influence the implementation of on-farm *Salmonella* control measures by pig farmers in England. *Preventive Veterinary Medicine*, *133*, 22-30. <https://doi.org/10.1016/j.prevetmed.2016.09.009>

Merrill, S. C., Moegenburg, S., Koliba, C. J., Zia, A., Trinity, L., Clark, E., Bucini, G., Wiltshire, S., Sellnow, T., Sellnow, D., & Smith, J. M. (2019). Willingness to comply with biosecurity in livestock facilities: Evidence from experimental simulations. *Frontiers in Veterinary Science*, *6*, 156. <https://doi.org/10.3389/fvets.2019.00156>

Merrill, S. C., Trinity, L., Clark, E. M., Shrum, T. R., Koliba, C. J., Zia, A., Bucini, G., Sellnow, T. L., Sellnow, D. D., & Smith, J. M. (2021). Message delivery strategy influences willingness to comply with biosecurity. *Frontiers in Veterinary Science*, *8*, 667265. <https://doi.org/10.3389/fvets.2021.667265>

Nampanya, S., Suon, S., Rast, L., & Windsor, P. A. (2012). Improvement in smallholder farmer knowledge of cattle production, health and biosecurity in Southern Cambodia between 2008 and 2010. *Transboundary and Emerging Diseases*, *59*(2), 117-127. <https://doi.org/10.1111/j.1865-1682.2011.01247.x>

Raasch, S., Collineau, L., Postma, M., Backhans, A., Sjolund, M., Belloc, C., Emanuelson, U., Beilage, E. G., Stark, K., Dewulf, J., & on the behalf of the, M. C. (2020). Effectiveness of alternative measures to reduce antimicrobial usage in pig production in four European countries. *Porcine Health Management*, *6*, 6. <https://doi.org/10.1186/s40813-020-0145-6>

Racicot, M., Cardinal, A. M., Tremblay, D., & Vaillancourt, J.-P. (2022). Technologies monitoring and improving biosecurity compliance in barn anterooms. *Frontiers in Veterinary Science*, *9*, Article 1005144. <https://doi.org/10.3389/fvets.2022.1005144>

Racicot, M., Venne, D., Durivage, A., & Vaillancourt, J.-P. (2012). Evaluation of strategies to enhance biosecurity compliance on poultry farms in Quebec: effect of audits and cameras. *Preventive Veterinary Medicine*, *103*(2-3), 208-218. <https://doi.org/10.1016/j.prevetmed.2011.08.004>

Rimi, N. A., Sultana, R., Ishtiak-Ahmed, K., Rahman, M. Z., Hasin, M., Islam, M. S., Azziz-Baumgartner, E., Nahar, N., Gurley, E. S., & Luby, S. P. (2016). Understanding the failure of a behavior change intervention to reduce risk behaviors for avian influenza transmission among backyard poultry raisers in rural Bangladesh: a focused ethnography. *BMC Public Health*, *16*(1), 858. <https://doi.org/10.1186/s12889-016-3543-6>

Schreuder, J., Simitopoulou, M., Angastiniotis, K., Ferrari, P., Wolthuis-Fillerup, M., Kefalas, G., & Papasolomontos, S. (2023). Development and implementation of a risk assessment tool for broiler farm biosecurity and a health intervention plan in the Netherlands, Greece, and Cyprus [Article]. *Poultry Science*, *102*(2), Article 102394. <https://doi.org/10.1016/j.psj.2022.102394>

Scollo, A., Levallois, P., Fourichon, C., Motta, A., Mannelli, A., Lombardo, F., & Ferrari, P. (2022). Monitoring means and results of biosecurity in pig fattening farms: Systematic assessment of measures in place and exploration of biomarkers of interest. *Animals*, *12*(19), Article 2655. <https://doi.org/10.3390/ani12192655>

Scollo, A., Perrucci, A., Stella, M. C., Ferrari, P., Robino, P., & Nebbia, P. (2023). Biosecurity and hygiene procedures in pig farms: Effects of a tailor-made approach as monitored by environmental samples. *Animals*, *13*(7), Article 1262. <https://doi.org/10.3390/ani13071262>

Smith, M. H., Meehan, C. L., & Borba, J. A. (2014). Bio-Security proficiencies project for beginning producers in 4-H. *Journal of Extension*, *52*(6), Article 2. <https://doi.org/10.34068/joe.52.06.02>

Tilli, G., Laconi, A., Galuppo, F., Grilli, G., Zbikowski, A., Amalraj, A., & Piccirillo, A. (2024). Supporting measures to improve biosecurity within Italian poultry production. *Animals*, *14*(12), Article 1734. <https://doi.org/10.3390/ani14121734>

Trinity, L., Merrill, S. C., Clark, E. M., Koliba, C. J., Zia, A., Bucini, G., & Smith, J. M. (2020). Effects of social cues on biosecurity compliance in livestock facilities: Evidence from experimental simulations. *Frontiers in Veterinary Science*, *7*, Article 130. <https://doi.org/10.3389/fvets.2020.00130>
