# Supplementary material for: How can we change behavior to bolster biosecurity? A scoping review and recommendations
Source: Front Vet Sci. 2026 Jul 22;13:1851630. doi: 10.3389/fvets.2026.1851630 (PMC13437398; doi:10.3389/fvets.2026.1851630)
Supplement: Supplementary file 5 [file Table_5.docx]

Supplementary Table 5

Intervention component mapping of studies designed to change behavior at the production or management level to protect animal health.

| Index | Change Target | | Intervention Approach | Intervention Format | Intervention Components | Intervention Functions (BCW) | Mapping to COM-B | Reference |
| --- | --- | --- | --- | --- | --- | --- | --- | --- |
| 1 | Knowledge, behavior | | Education, instruction, assessment | Online (self-learning), printable materials, online (self-assessment) | Online educational modules, infographics and signs, quizzes | Questioning/Observing; Education; (indirectly Training; Persuasion; Environmental Restructuring) | C – psychological, M – reflective (and automatic) | (Agrawal et al., 2023) |
| 2 | Knowledge, behavior | | Assessment, advice | In-person, one-on-one | BSM audit questionnaire, specific advice | Questioning/Observing; Education; Persuasion | C – physical,  M – reflective (and automatic) | (Ahmed et al., 2021) |
| 3 | Knowledge, motivation | | Education and training, education, assessment and advice | In-person, group; printed materials; self-assessment | Workshop; fact sheets, self-assessment of risky practices and advice | Education; Training; Questioning/Observing; Persuasion | C – psychological, M – reflective (and automatic) | (Allen et al., 2019) |
| 4 | Behavior, attitude, motivation | | Assessment, planning, coaching | In-person, one-on-one | Attitude survey and BSM audit questionnaire, action plan, monitoring | Questioning/Observing; Education; Planning; Persuasion | M – reflective | (Amalraj et al., 2024) |
| 5 | Knowledge, motivation, health outcomes | | Assessment, advice, goal setting | In-person, one-on-one | Risk assessment, tailored advice, commitment, seroprevalence | Questioning/Observing; Education; Persuasion | C – psychological, M – reflective | (Cardwell et al., 2016) |
| 6 | Knowledge, attitude, motivation | | Education, planning, commitment, assessment | In-person, group participation | Presentation on ASF, focus group discussions, community contract, group assessment of implementation | Questioning/Observing; Education; Persuasion; Environmental Restructuring | C – psychological; O – physical and social; M – reflective | (Chenais et al., 2023) |
| Index | Change Target | | Intervention Approach | Intervention Format | Intervention Components | Intervention Functions (BCW) | Mapping to COM-B | Reference |
| 7 | Knowledge | | Education | Online, media | Website | Education | C – psychological;  M – reflective | (Colvin et al., 2021) |
| 8 | Health outcomes | | Training, education | In-person, group meetings | Training of trainers, education of farmers, seroprevalence and mortality rates | Training (of trainers); Education (of farmers); Persuasion; Environmental Restructuring; Enablement | C – psychological & physical ;  O – social; M – reflective | (Conan, Luce Goutard, et al., 2013) reported on health outcomes; (Conan, Ponsich, et al., 2013) reported on knowledge and behavior changes. |
| 10 | Knowledge, attitude, skill | | Education, assessment, training | In-person, group | Attitude questionnaires, training exercises, application of knowledge | Questioning/Observing; Training; Education; Persuasion | C – psychological & physical;  O – social; M – reflective | (Delpont et al., 2024) |
| 11 | | Knowledge, attitude, skill | Education, assessment, training | In-person, group | Co-created training, trainings, KAP surveys, poster | Training; Education; Persuasion; Enablement; Questioning/Observing | C – psychological & physical;  O – social; M – reflective | (Dione et al., 2020) |
| 12 | | Knowledge, behavior | Education, assessment | Online, media | Communication campaign, surveys | Questioning/Observing; Education; Persuasion | C – psychological & physical | (Gillespie et al., 2021) |
| 13 | | Knowledge, attitude, motivation, behavior | Assessment, advice | In-person, one-on-one | KAP surveys, tailored advice, advice cards | Questioning/Observing; Education; Persuasion; Enablement | C – psychological; M – reflective | (Gosling et al., 2014) |
| 14 | | Behavior | Communication | Environment (online simulation) | Risk messages and methods | Education; Persuasion; Incentivization | M – reflective and automatic | (Liu et al., 2022) |
| 15a, 15b | | Knowledge, attitude, skill | Education, assessment, training | In-person, train-the-trainer | Formal and on-the-job training, KAP survey | Training; Education; Enablement | C – psychological & physical;  M – reflective;  O – social | (MacPhillamy et al., 2022) |
| Index | Change Target | | Intervention Approach | Intervention Format | Intervention Components | Intervention Functions (BCW) | Mapping to COM-B | Reference |
| 16 | | Knowledge, skill | Education, training, assessment | In-person, train-the-trainer, tech transfer | Training, tech transfer and applied research | Training; Education; Enablement; Questioning/Observing | C – psychological & physical;  O – social; M – automatic | (Nampanya et al., 2012) |
| 17a, 17b | | Behavior | Feedback | Environment (monitoring technology) | RFID monitoring of hygiene, with or without alarm | Environmental Restructuring; Education (b): Persuasion; Coercion?; Questioning/Observing | M - reflective | (Racicot et al., 2022) |
| 18 | | Knowledge, behavior | Monitoring | Environment (monitoring technology), in-person (audit) | Poster, visible camera, audits | Environmental Restructuring; Persuasion; Coercion?; Questioning/Observing | C – psychological; M – reflective | (Racicot et al., 2012) |
| 20 | | Knowledge, behavior, health outcomes | Assessment, planning, review | In-person, one-on-one | BSM audit questionnaire, co-creation of plan, repeat of audit | Questioning/Observing; Planning; Education; Persuasion; Enablement | C – psychological & physical;  O – social & physical;  M – reflective | (Schreuder et al., 2023) |
| 21 | | Knowledge, behavior, health outcomes | Assessment, planning, review | In-person, one-on-one | BSM audit questionnaire, co-creation of plan, repeat of audit | Education; Persuasion; Planning; Environmental Restructuring; Questioning/Observing; Enablement | C – psychological & physical;  O – social & physical;  M – reflective | (Scollo et al., 2022) |
| 22a | | Knowledge, skill | Education, training | 4-H project | Educational modules, documentation of learning, public presentation | Education; Training; Enablement | C – psychological & physical | (Smith et al., 2014) |
| Index | Change Target | | Intervention Approach | Intervention Format | Intervention Components | Intervention Functions (BCW) | Mapping to COM-B | Reference |
| 22b | | Knowledge | Education | In-person, group | Attended public presentation | Education; Persuasion | C – psychological | (Smith et al., 2014) |
| 23a | | Motivation | Elicitation | In-person, interviews, group discussion | Identify supportive measures to pilot | Questioning/Observing; Planning | O – social;  M – reflective | (Tilli et al., 2024) |
| 23b | | Attitudes, knowledge, motivation, behavior | Assessment, planning, coaching | In-person, one-on-one | Attitude survey, BSM audit questionnaire, goal setting, coaching | Questioning/Observing; Education; Persuasion; Planning; Environmental Restructuring; Enablement | C – psychological;  O – physical and social; M – reflective | (Tilli et al., 2024) |
| 24 | | Behavior | Communication, environment | Environment (online simulation) | Risk messages and methods and social cues | Education; Persuasion; Environmental Restructuring (or Modeling); Incentivization | M – reflective and automatic;  O – social | (Trinity et al., 2020) |
| 25 | | Knowledge, skills, behavior | Education, training | In-person, train-the-trainer, environment | Training of trainers, training and tech support of farmers, model farms | Education; Training; Enablement; Modeling | C – psychological & physical;  O – social & physical;  M – reflective | (Bhandari et al., 2011) |
| 26 | | Behavior | Assessment, planning, commitment, review, compensation | In-person, with advisory group | Assess options, co-create plan, commit/contract, assess impact | Questioning/Observing; Education; Persuasion; Enablement; Planning; Environmental Restructuring | C – psychological & physical;  O – social & physical;  M – reflective | (Collineau et al., 2017) This report is from same study as (Raasch et al., 2020). |
| 28a | | Knowledge, behavior | Assessment, planning, prompts | One-on-one, mailing | Assess options, co-create plan, prompts | Questioning/Observing; Planning; Education; Persuasion; Enablement | C – psychological & physical;  O – social & physical;  M – reflective | (Grant et al., 2018) |
| Index | Change Target | | Intervention Approach | Intervention Format | Intervention Components | Intervention Functions (BCW) | Mapping to COM-B | Reference |
| 28b | | Knowledge, behavior | Assessment, training | Group meetings | Group training, message framing, survey | Questioning/Observing; Education; Persuasion | C – psychological & physical;  O – social & phys.;  M – reflective | (Grant et al., 2018) |
| 28c | | Knowledge, behavior | Assessment, education | Mailings | Mailings, message framing, survey | Questioning/Observing; Education; Persuasion | C – psychological & physical;  O – social & phys.;  M – reflective | (Grant et al., 2018) |
| 29 | | Policy | Economic analysis | Indirect | Analyzed production contracts | Incentivization | M – reflective;  O – physical | (Komaladara et al., 2018) |
| 30 | | Behavior, intentions, motivation | Model farms | Environment | Model farms, surveys of determinants | Modeling | C – psychological & physical;  O – social & phys.;  M – reflective | (Marier et al., 2016) |
| 31a, 31b | | Behavior | Communication | Environment (online simulation) | Risk messages and methods | Education; Persuasion; Incentivization | M – reflective and automatic | (Merrill et al., 2019) |
| 32 | | Behavior | Communication | Environment (online simulation) | Risk messages and methods | Education; Persuasion; Incentivization | M – reflective and automatic | (Merrill et al., 2021) |
| 34 | | Awareness, knowledge, behavior | Education, training | Group meetings, environment | Tailored messages, posters | Questioning/Observing; Education | C – psychological & physical;  O – social & phys.;  M – reflective | (Rimi et al., 2016) |
| 35 | | Behavior | Assessment, training, planning | Group meetings and one-on-one | BSM audit questionnaire, plan co-creation, monitoring | Education; Persuasion; Planning; Environmental Restructuring; Questioning/Observing; Enablement | C – psychological & physical;  O – social & phys.;  M – reflective | (Scollo et al., 2023) |

4-H = an American youth development program founded on the principle of learning by doing; BSM = biosecurity measure; C = capability; O = opportunity; M = motivation

References

Agrawal, I., Bromfield, C., & Varga, C. (2023). Assessing and improving on-farm biosecurity knowledge and practices among swine producers and veterinarians through online surveys and an educational website in Illinois, United States. *Frontiers in Veterinary Science*, *10*, Article 1167056. <https://doi.org/10.3389/fvets.2023.1167056>

Ahmed, H. A., Mohamed, M. E. M., Erfan, A. M., Abdelkarim, L., & Awadallah, M. A. I. (2021). Investigating the biosecurity measures' applications in poultry farms and its relationship with the occurence of avian influenza. *Slovenian Veterinary Research*, *58*, 315-321. <https://doi.org/10.26873/SVR-1451-2021>

Allen, A. J., Neibergs, J. S., Llewellyn, D. A., Smith, S. M., Kerr, S. R., Moore, D. A., & Hudson, T. D. (2019). Developing herd health education for and assessing risky practices of cow-calf producers. *Journal of Extension*, *57*(5), Article 16. <https://doi.org/10.34068/joe.57.05.16>

Amalraj, A., Van Meirhaeghe, H., Chantziaras, I., & Dewulf, J. (2024). Assessing the impact of on-farm biosecurity coaching on farmer perception and farm biosecurity status in Belgian poultry production. *Animals*, *14*(17), Article 2498. <https://doi.org/10.3390/ani14172498>

Bhandari, D. P., Wollen, T. S., & Lohani, M. N. (2011). Preventing highly pathogenic avian influenza (HPAI) at the rural community level: a case study from Cambodia. *Tropical Animal Health and Production*, *43*(6), 1071-1073. <https://doi.org/10.1007/s11250-011-9828-y>

Cardwell, J. M., Van Winden, S., Beauvais, W., Mastin, A., De Glanville, W. A., Hardstaff, J., Booth, R. E., Fishwick, J., & Pfeiffer, D. U. (2016). Assessing the impact of tailored biosecurity advice on farmer behaviour and pathogen presence in beef herds in England and Wales. *Preventive Veterinary Medicine*, *135*, 9-16. <https://doi.org/10.1016/j.prevetmed.2016.10.018>

Chenais, E., Fischer, K., Aliro, T., Ståhl, K., & Lewerin, S. S. (2023). Co-created community contracts support biosecurity changes in a region where African swine fever is endemic-Part II: Implementation of biosecurity measures. *Preventive Veterinary Medicine*, *214*, Article 105902. <https://doi.org/10.1016/j.prevetmed.2023.105902>

Collineau, L., Rojo-Gimeno, C., Leger, A., Backhans, A., Loesken, S., Nielsen, E. O., Postma, M., Emanuelson, U., Beilage, E. G., Sjolund, M., Wauters, E., Stark, K. D. C., Dewulf, J., Belloc, C., & Krebs, S. (2017). Herd-specific interventions to reduce antimicrobial usage in pig production without jeopardising technical and economic performance. *Preventive Veterinary Medicine*, *144*, 167-178. <https://doi.org/10.1016/j.prevetmed.2017.05.023>

Colvin, A. F., Reeve, I., Thompson, L. J., Kahn, L. P., & Walkden-Brown, S. W. (2021). Australian surveys on parasite control in sheep between 2003 and 2019 reveal marked regional variation and increasing utilisation of online resources and on-farm biosecurity practices [Article]. *Veterinary Parasitology: Regional Studies and Reports*, *25*, Article 100614. <https://doi.org/10.1016/j.vprsr.2021.100614>

Conan, A., Luce Goutard, F., Holl, D., Ra, S., Ponsich, A., Tarantola, A., Sorn, S., & Vong, S. (2013). Cluster randomised trial of the impact of biosecurity measures on poultry health in backyard flocks. *Veterinary Journal*, *198*(3), 649-655. <https://doi.org/10.1016/j.tvjl.2013.09.010>

Conan, A., Ponsich, A., Luce Goutard, F., Khiev, R., Tarantola, A., Sorn, S., & Vong, S. (2013). A community-based education trial to improve backyard poultry biosecurity in rural Cambodia. *Acta Tropica*, *125*(3), 294-302. <https://doi.org/10.1016/j.actatropica.2012.12.006>

Delpont, M., Chavoix, C., Fourtune, L., Durivage, A., Guérin, J. L., Vaillancourt, J. P., & Paul, M. C. (2024). Making the invisible visible: Effectiveness of a training program to increase awareness and biosecurity compliance in poultry barn anterooms, using glowing substances [Article]. *Preventive Veterinary Medicine*, *233*, Article 106349. <https://doi.org/10.1016/j.prevetmed.2024.106349>

Dione, M. M., Dohoo, I., Ndiwa, N., Poole, J., Ouma, E., Amia, W. C., & Wieland, B. (2020). Impact of participatory training of smallholder pig farmers on knowledge, attitudes and practices regarding biosecurity for the control of African swine fever in Uganda. *Transboundary and Emerging Diseases*, *67*(6), 2482-2493. <https://doi.org/10.1111/tbed.13587>

Gillespie, A. V., Carter, S. D., Blowey, R. W., Staton, G. J., Walsh, T. R., & Evans, N. J. (2021). Measuring the impact of bovine digital dermatitis research on knowledge and practice of biosecurity during cattle foot-trimming. *Journal of Dairy Research*, *88*(1), 60-63, Article Pii s0022029921000170. <https://doi.org/10.1017/S0022029921000170>

Gosling, R. J., Martelli, F., Wintrip, A., Sayers, A. R., Wheeler, K., & Davies, R. H. (2014). Assessment of producers' response to Salmonella biosecurity issues and uptake of advice on laying hen farms in England and Wales. *British Poultry Science*, *55*(5), 559-568. <https://doi.org/10.1080/00071668.2014.949620>

Grant, C., Kaler, J., Ferguson, E., O'Kane, H., & Green, L. E. (2018). A comparison of the efficacy of three intervention trial types: postal, group, and one-to-one facilitation, prior management and the impact of message framing and repeat messages on the flock prevalence of lameness in sheep. *Preventive Veterinary Medicine*, *149*, 82-91. <https://doi.org/10.1016/j.prevetmed.2017.11.013>

Komaladara, A., Patrick, I., & Hoang, N. (2018). Contract bonus systems to encourage biosecurity adoption on small-scale broiler farms in Indonesia. *Animal Production Science*, *58*(3), 595-600. <https://doi.org/10.1071/AN15845>

Liu, T.-L., Merrill, S. C., O'Keefe, A., Clark, E. M., Langle-Chimal, O. D., Trinity, L., Shrum, T. R., Koliba, C., Zia, A., Sellnow, T. L., Sellnow, D. D., & Smith, J. M. (2022). Effects of message delivery on cross-cultural biosecurity compliance: Insights from experimental simulations. *Frontiers in Veterinary Science*, *9*, Article 984945. <https://doi.org/10.3389/fvets.2022.984945>

MacPhillamy, I., Olmo, L., Young, J., Nampanya, S., Suon, S., Khounsy, S., Windsor, P., Toribio, J.-A., & Bush, R. (2022). Changes in farmer animal health and biosecurity knowledge, attitudes and practices: Insights from Cambodia and Laos. *Transboundary and Emerging Diseases*, *69*(4), e517-e531. <https://doi.org/10.1111/tbed.14328>

Marier, E., Piers Smith, R., Ellis-Iversen, J., Watson, E., Armstrong, D., Hogeveen, H., & Cook, A. J. (2016). Changes in perceptions and motivators that influence the implementation of on-farm *Salmonella* control measures by pig farmers in England. *Preventive Veterinary Medicine*, *133*, 22-30. <https://doi.org/10.1016/j.prevetmed.2016.09.009>

Merrill, S. C., Moegenburg, S., Koliba, C. J., Zia, A., Trinity, L., Clark, E., Bucini, G., Wiltshire, S., Sellnow, T., Sellnow, D., & Smith, J. M. (2019). Willingness to comply with biosecurity in livestock facilities: Evidence from experimental simulations. *Frontiers in Veterinary Science*, *6*, 156. <https://doi.org/10.3389/fvets.2019.00156>

Merrill, S. C., Trinity, L., Clark, E. M., Shrum, T. R., Koliba, C. J., Zia, A., Bucini, G., Sellnow, T. L., Sellnow, D. D., & Smith, J. M. (2021). Message delivery strategy influences willingness to comply with biosecurity. *Frontiers in Veterinary Science*, *8*, 667265. <https://doi.org/10.3389/fvets.2021.667265>

Nampanya, S., Suon, S., Rast, L., & Windsor, P. A. (2012). Improvement in smallholder farmer knowledge of cattle production, health and biosecurity in Southern Cambodia between 2008 and 2010. *Transboundary and Emerging Diseases*, *59*(2), 117-127. <https://doi.org/10.1111/j.1865-1682.2011.01247.x>

Raasch, S., Collineau, L., Postma, M., Backhans, A., Sjolund, M., Belloc, C., Emanuelson, U., Beilage, E. G., Stark, K., Dewulf, J., & on the behalf of the, M. C. (2020). Effectiveness of alternative measures to reduce antimicrobial usage in pig production in four European countries. *Porcine Health Management*, *6*, 6. <https://doi.org/10.1186/s40813-020-0145-6>

Racicot, M., Cardinal, A. M., Tremblay, D., & Vaillancourt, J.-P. (2022). Technologies monitoring and improving biosecurity compliance in barn anterooms. *Frontiers in Veterinary Science*, *9*, Article 1005144. <https://doi.org/10.3389/fvets.2022.1005144>

Racicot, M., Venne, D., Durivage, A., & Vaillancourt, J.-P. (2012). Evaluation of strategies to enhance biosecurity compliance on poultry farms in Quebec: effect of audits and cameras. *Preventive Veterinary Medicine*, *103*(2-3), 208-218. <https://doi.org/10.1016/j.prevetmed.2011.08.004>

Rimi, N. A., Sultana, R., Ishtiak-Ahmed, K., Rahman, M. Z., Hasin, M., Islam, M. S., Azziz-Baumgartner, E., Nahar, N., Gurley, E. S., & Luby, S. P. (2016). Understanding the failure of a behavior change intervention to reduce risk behaviors for avian influenza transmission among backyard poultry raisers in rural Bangladesh: a focused ethnography. *BMC Public Health*, *16*(1), 858. <https://doi.org/10.1186/s12889-016-3543-6>

Schreuder, J., Simitopoulou, M., Angastiniotis, K., Ferrari, P., Wolthuis-Fillerup, M., Kefalas, G., & Papasolomontos, S. (2023). Development and implementation of a risk assessment tool for broiler farm biosecurity and a health intervention plan in the Netherlands, Greece, and Cyprus [Article]. *Poultry Science*, *102*(2), Article 102394. <https://doi.org/10.1016/j.psj.2022.102394>

Scollo, A., Levallois, P., Fourichon, C., Motta, A., Mannelli, A., Lombardo, F., & Ferrari, P. (2022). Monitoring means and results of biosecurity in pig fattening farms: Systematic assessment of measures in place and exploration of biomarkers of interest. *Animals*, *12*(19), Article 2655. <https://doi.org/10.3390/ani12192655>

Scollo, A., Perrucci, A., Stella, M. C., Ferrari, P., Robino, P., & Nebbia, P. (2023). Biosecurity and hygiene procedures in pig farms: Effects of a tailor-made approach as monitored by environmental samples. *Animals*, *13*(7), Article 1262. <https://doi.org/10.3390/ani13071262>

Smith, M. H., Meehan, C. L., & Borba, J. A. (2014). Bio-Security proficiencies project for beginning producers in 4-H. *Journal of Extension*, *52*(6), Article 2. <https://doi.org/10.34068/joe.52.06.02>

Tilli, G., Laconi, A., Galuppo, F., Grilli, G., Zbikowski, A., Amalraj, A., & Piccirillo, A. (2024). Supporting measures to improve biosecurity within Italian poultry production. *Animals*, *14*(12), Article 1734. <https://doi.org/10.3390/ani14121734>

Trinity, L., Merrill, S. C., Clark, E. M., Koliba, C. J., Zia, A., Bucini, G., & Smith, J. M. (2020). Effects of social cues on biosecurity compliance in livestock facilities: Evidence from experimental simulations. *Frontiers in Veterinary Science*, *7*, Article 130. <https://doi.org/10.3389/fvets.2020.00130>
